# Single-cell transcriptomics of the myeloid milieu reveals an angiogenic niche in triple-negative breast cancer

**DOI:** 10.1038/s12276-025-01571-5

**Published:** 2025-11-07

**Authors:** Yechan Choi, Minkyu Shim, Suhn Hyung Kim, Duk Ki Kim, Juhee Jeong, Jinyoung Byeon, Giyong Jang, Ji-Yeon Kim, Paul Robson, Charles Lee, Han-Byoel Lee, Keehoon Jung

**Affiliations:** 1 Department of Anatomy and Cell Biology, Department of Biomedical Sciences, Seoul National University College of Medicine, Seoul, Republic of Korea; 2 Institute of Allergy and Clinical Immunology, Seoul National University Medical Research Center, Seoul, Republic of Korea; 3 Department of Surgery, Seoul National University College of Medicine, Seoul, Republic of Korea; 4 Cancer Research Institute, Seoul National University, Seoul, Republic of Korea; 5 Biomedical Research Institute, Seoul National University Hospital, Seoul, Republic of Korea; 6 Genomic Medicine Institute, Medical Research Center, Seoul National University, Seoul, Republic of Korea; 7 Ewha Biomedical Research Institute, Ewha Womans University Medical Center, Seoul, Republic of Korea; 8 Division of Hematology-Oncology, Department of Medicine, Samsung Medical Center, Sungkyunkwan University School of Medicine, Seoul, Republic of Korea; 9 The Jackson Laboratory for Genomic Medicine, Farmington, CT USA

**Keywords:** Tumour immunology, Cancer microenvironment, Breast cancer

## Abstract

Intratumoral myeloid cells are highly heterogeneous in terms of development and function and are pivotal for forming and regulating the tumor microenvironment. However, the myeloid milieu in triple-negative breast cancer (TNBC) remains poorly understood. Here, to elucidate this myeloid milieu, we integrated in-house and public single-cell RNA sequencing data. We detected diverse neutrophil and mononuclear-phagocyte subtypes and delineated their developmental trajectories and functions. Of particular interest were the *VEGFA*^*hi*^ neutrophil and *SPP1*^*hi*^ macrophage subtypes, which displayed protumoral functions, including angiogenesis. Spatial transcriptomics revealed that they colocalized with epithelial cancer cells and *APLN*^*hi*^ endothelial tip cells in a hypoxic region forming an angiogenic niche. Moreover, patients with *SPP1*^*hi*^ macrophage-enriched TNBC showed poor prognosis, which worsened in patients who also displayed abundant *VEGFA*^*hi*^ neutrophils. These subtypes were also conserved in multiple murine TNBC models. This comprehensive analysis of the myeloid population in TNBC thus reveals a previously undercharacterized interaction between *VEGFA*^*hi*^ neutrophils and *SPP1*^*hi*^ macrophages, elucidating their contributions in the formation of an angiogenic niche.

## Unraveling myeloid interactions in triple-negative breast cancer

Breast cancer is a common cancer and a leading cause of cancer-related deaths. Triple-negative breast cancer (TNBC) is particularly challenging to treat due to the lack of specific targets for therapy. This study explores the role of myeloid cells in TNBC. The researchers used single-cell RNA sequencing to analyze myeloid cells from TNBC samples. They identified various subtypes of these cells, including neutrophils and macrophages, which have different roles in the tumor environment. The study found that certain subtypes, such as SPP1^hi^ macrophages and VEGFA^hi^ neutrophils, colocalize in the tumor microenvironment and participate in promoting blood vessel formation, leading to poor patient outcomes. The findings suggest that targeting these specific myeloid cell subtypes could improve treatment for TNBC. Future research may focus on developing therapies that specifically target these protumoral cells to enhance patient survival.

This summary was initially drafted using artificial intelligence, then revised and fact-checked by the author.

## Introduction

Breast cancer is not only the most frequently diagnosed cancer, but also a leading cause of cancer-related deaths^1^. In particular, triple-negative breast cancer (TNBC), which accounts for 15–20% of all breast cancer cases, is associated with poor prognosis^2,3^. This reflects the absence or low expression of standard molecular targets—namely, the estrogen receptor (ER), progesterone receptor (PR) and human epidermal growth factor receptor 2 (HER2)—which limits available treatment options^4^.

The tumor microenvironment (TME) includes diverse immune cells^5,6^. Myeloid cells are particularly important because of their involvement in several protumoral processes. For instance, neutrophils often promote tumor progression, and their accumulation in breast tumors has been linked to therapeutic resistance^7^. Tumor-associated macrophages (TAMs) are known to promote angiogenesis, tumor recurrence and metastasis^8–11^. Moreover, TAMs recruit immunosuppressive leukocytes and hinder cytotoxic T cell function, fostering a protumoral environment^12–14^. However, many strategies targeting the myeloid milieu have been ineffective^15,16^. The key reason for this inefficacy is the oversimplification of their heterogeneity, which includes both protumoral and antitumoral subtypes^13,17^.

Single-cell RNA sequencing (scRNA-seq) has enabled the comprehensive understanding of the complex TME at single-cell resolution^18–20^. Moreover, spatial transcriptomics provides insights into the spatial context and identifies functional niches^21^. While myeloid cells have been studied across various cancer types—such as colon, pancreatic, lung, and liver cancers, as well as in pan-cancer studies—the myeloid landscape in TNBCs remains underexplored^19,22–24^. Previous scRNA-seq studies focusing on breast cancer have often failed to distinguish TNBC from other subtypes (non-TNBC), yielding only limited insights into the myeloid landscape within TNBC^25,26^.

Here, we investigated the diverse functions and developmental trajectories of the myeloid cells in TNBC. In particular, our study captures neutrophils, which are often underrepresented in scRNA-seq studies, revealing several subtypes and two major trajectories. We also delineated multiple macrophage subtypes of different origins and functions, focusing on the protumoral *SPP1*^*hi*^ Mφ subset. Spatial transcriptomics further revealed an angiogenic niche characterized by an accumulation of *VEGFA*^*hi*^ neutrophils and *SPP1*^*hi*^ Mφs in proximity to *APLN*^*hi*^ endothelial tip cells. Bulk deconvolution revealed the clinical relevance of both *VEGFA*^*hi*^ neutrophils and *SPP1*^*hi*^ Mφs. Finally, we validated that *SPP1*^*hi*^ Mφs were conserved in multiple murine TNBC models, providing a foundation for further translational research.

## Methods

### Patient sample collection

This study was approved by the Seoul National University Hospital Institutional Review Board (IRB approval no. 1905-087-1034) and was conducted in accordance with the tenets of the Declaration of Helsinki. Fresh TNBC samples were obtained from patients who underwent surgical resection at Seoul National University Hospital. Patients with infectious or autoimmune diseases were not included in the cohort. Written informed consent was obtained from each patient. All patient data were anonymized (Supplementary Table 1).

### Cell culture

EO771, THP-1 and HL-60 cell lines were obtained from the American Type Culture Collection. The EO771 cell line was cultured in Dulbecco’s modified Eagle medium (L0103-500; Biowest) supplemented with 10% fetal bovine serum (FBS) (S1480-500; Biowest) and 1% antibiotic antimycotic solution (LS 203-01-100; Welgene) (complete media). THP-1 and HL-60 were cultured in RPMI-1640 (L0498-500; Biowest) supplemented with 10% FBS and 1% antibiotic antimycotic solution. To induce macrophage-like polarization, THP-1 cells were cultured in six-well plates by stimulating with 100 ng/ml phorbol-12-myristate-13-acetate (PMA) for 4 days, followed by a resting period of 24 h without PMA^27^. Neutrophil-like cells (HL-60N) were induced by culturing HL-60 cells in 1% dimethyl sulfoxide (DMSO) (D8418; Sigma-Aldrich) for 6 days^28^. Subsequently, both macrophage-like THP-1 and HL-60N cells were cultured in hypoxic conditions (1% O_2_) for 24 h. Human umbilical vein endothelial cells (ECs) were cultured on culture flasks coated with gelatin (G1890; Sigma-Aldrich) in Endothelial Cell Growth Medium-2 (EGM-2) (CC-3162; Lonza) supplemented with EGM-2 SingleQuots Supplements (CC-4176; Lonza).

### Bone marrow monocyte isolation and differentiation

Femur and tibia were obtained from WT mice and bone marrow cells were collected by flushing the bone marrow in RPMI-1640 containing 10% FBS and 1% antibiotic antimycotic solution. Erythrocytes were lysed by ACK lysis buffer (420301; BioLegend) followed by neutralization with complete media. The collected cells were seeded in six-well plates and differentiated into macrophages (bone marrow-derived macrophages, BMDMs) with 20 ng/ml M-CSF (315-02; Peprotech). Adherent cells were collected on day 7 and seeded at 1 × 10^6^ cells per well in six-well plates. Cells were incubated under either hypoxic (1% O_2_) or normoxic conditions.

### Orthotopic murine TNBC-model generation

To generate orthotopic TNBC models, 1 × 10^6^ EO771 tumor cells were resuspended in 50 µl complete media. Female 7-week-old C57BL/6 mice were anesthetized with ketamine (100 mg/kg) and xylazine (10 mg/kg) solution via intraperitoneal injection, and their bilateral second mammary fat pads were injected with the cell suspension. The tumors were resected 33 days after implantation.

### Tissue digestion, single-cell suspension and fluorescence-activated cell sorting

After surgery, TNBC samples were chopped into submilimeter particles manually and incubated for 30 min in a 37 °C 5% CO_2_ incubator with RPMI-1640 supplemented with 1 mg/ml hyaluronidase (H6254; Sigma), 1 mg/ml collagenase type IV (LS004189; Worthington Biochemical Corp.) and 0.5 mg/ml DNase I (DN25; Sigma). After tissue digestion, cells were filtered with 70-µm cell strainers (93070; SPL). Erythrocytes were lysed with 3 ml ACK lysis buffer on ice for 5 min. The reaction was stopped by adding 10 ml 1× PBS (PR4007-100-00; Biosesang). After centrifugation, cells were resuspended with 1 ml of RPMI-1640 supplemented with 10% FBS.

To isolate myeloid cells, single-cell suspensions were incubated with a Live/Dead fixable blue dead-stain kit (L34962; Thermo) and sequentially stained according to the manufacturer’s instructions with anti-human CD45 (clone: HI30; Alexa 700; BioLegend) and anti-human CD11b (clone: ICRF44; APC/Cy7; BioLegend) antibodies. Cell sorting was performed with a BD Aria III sorter. Sorted cells were immediately sent for scRNA-seq.

### Human bulk RNA-seq analysis

Publicly available human breast-cancer bulk RNA-seq data were acquired using TCGAbiolinks (v2.32.0)^29^. Public scRNA-seq data were first filtered to remove the genes that were expressed in <50 cells across every immune cell type from GSE176078^26^. The TNBCs and non-TNBCs in this cohort were defined by their annotated immunohistochemistry information, showing their ER, PR and HER2 status. The TNBCs and non-TNBCs were subjected to principal component analysis (PCA). The top 200 genes accounting for the first and second principal components, respectively, were subjected to Gene Ontology (GO) analysis using clusterProfiler (v4.12.2) and GO biological processes (GO: BP)^30,31^. The genes that were differentially expressed between TNBC and non-TNBC samples were determined with DESeq2 (v1.44.0)^32^. Genes with a baseMean greater than 10, an adjusted *P* value less than 0.05 and a log_2_ fold change greater than 0.5 were subjected to GO analysis.

### Single-cell RNA-seq data preprocessing

In-house scRNA-seq data were aligned and quantified on the reference genome GRCh38 or mm10 depending on species by using Cell Ranger toolkit (v3.1.0) provided by 10x Genomics. The processed data from Cell Ranger were used for downstream analysis. Cells that expressed <150 genes or >25% mitochondrial genes were removed. Genes that were detected in fewer than three cells were excluded. To filter out doublets, DoubletFinder (v2.0.4) was applied to each sample^33^. The multiplet rates were estimated based on the 10X Genomics user guide.

For publicly available scRNA-seq data from GSE169246^34^, GSE176078^26^ and GSE195665^35^, the same protocol was applied to the count matrix. The clinical status of each sample was obtained from the original studies.

The filtered data were then log-normalized using the normalize_total and log1p functions in Scanpy (v1.9.8)^36^.

### Batch-effect correction and cluster annotation

We identified the highly variable genes (HVGs) that were conserved across samples using the scanpy.pl.highly_variable_genes function with the batch key assigned on the basis of the cell number of each sample. Parameters were adjusted to set the cutoffs for means and normalized dispersions, targeting sufficient number of genes for integration. Subsequently, PCA was performed using the scanpy.tl.pca function, with the top 50 components conserved for downstream analysis.

We then used the BBKNN package (v1.6.0) for batch correction, which identifies the nearest neighbors of each cell from each batch separately^37^. The bbknn.bbknn function was applied, using the same batch key used in the HVG calculation. We visualized the integration result by Uniform Manifold Approximation and Projection (UMAP), namely via the scanpy.tl.umap function.

Despite strict quality control, we found contamination of nonimmune cells. Thus, we used in silico fluorescence-activated cell sorting and unsupervised clustering to isolate myeloid cells based on canonical markers and previous conventions^38^. The resulting high-quality myeloid cells were then reintegrated following the same procedure.

We subsequently performed two rounds of unsupervised clustering using the scanpy.tl.leiden function with the resolution varying between 0.5 and 1.2. The first round of clustering distinguished monocytes/macrophages, neutrophils, dendritic cells (DCs) and plasmacytoid DCs (pDCs) using canonical markers. The monocytes/macrophages, neutrophils and DCs were then subjected to a second round of clustering to elucidate their heterogeneity. The differentially expressed genes (DEGs) in each cluster were determined using scanpy.tl.rank_genes_groups with the parameter setting ‘method=wilcoxon’. Ribosomal and mitochondrial genes were excluded, and those detected below 10% within the cluster were filtered using scanpy.tl.filter_rank_genes_groups. We determined and annotated the cell clusters based on their DEGs and previous conventions^23,25,39–45^. The ranking of the DEGs for downstream analysis was determined according to

$${{\mathrm{ranking}}}=(-\log \left({{\mathrm{adjusted}}}\,P\,{{\mathrm{value}}}\right))\times \log_2\,{{\mathrm{fold}\,}{\mathrm{change}}}.$$

For further analyses that required cell types beyond the myeloid milieu, such as bulk deconvolution and spatial deconvolution, we constructed a reference database by combining nonmyeloid cells from TNBC samples in GSE176078^26^ with our myeloid landscape.

### Differential expression analysis

Differential expression analysis between the SPP1 lineage and the classical lineage was performed using the Python package diffxpy (v0.7.4; theislab/diffxpy, https://github.com/theislab/diffxpy). Genes detected in <50 cells from each state were excluded before analysis. Here, we performed Wald tests using the de.test.wald function. We also included size factors that were calculated using Scran (v1.30.2)^46^. Multiple testing correction was conducted with the Benjamini–Hochberg method. Genes that expressed mean values >1.0 were preserved for downstream analysis.

### Functional analysis by GSEA and GSVA

Gene set enrichment analysis (GSEA) was conducted with Python package GSEApy (v1.0.6)^47^ by applying gseapy.prerank to the genes and their rankings. To compare conventional neutrophils with *CD74*^*hi*^ neutrophils, we used GO:BP as the background pathway. To compare the SPP1 lineage with the classical lineage, we used Hallmark as the background pathway. Pathways with false discovery rate <0.25 were considered significant, as recommended by the GSEA User Guide provided by MSigDB.

We applied R package GSVA (v3.9) to count the expression matrix for gene set variation analysis (GSVA)^48^. The pathways were selected from curated gene sets (C2), ontology gene sets (C5) and hallmark gene sets (H) in human MSigDB collections based on myeloid function and previous research^28,39^. For murine GSVA analysis, the corresponding pathways were obtained from mouse MSigDB collections.

### Trajectory analysis

Before trajectory analysis, the origin of the cells was carefully investigated. This led us to exclude proliferating and tissue-resident macrophages^25,42^. To better infer the trajectories, we also calculated diffusion maps, which implicitly align the cells on the basis of their developmental pathway. For calculation, scanpy.tl.diffmap was used under default parameters. Fourteen diffusion components were obtained for downstream analysis.

We performed trajectory analysis with R package slingshot (v2.12.0) and Monocle 3 (v2.32.0), as well as Python package scTour (v1.0.0)^49–51^. Initially, we reintegrated the non-TRMs and used scTour with HVGs adopted from the integration process. Default settings were used. The constructed vector fields were presented on recalculated UMAP reductions. We also analyzed the trajectories with slingshot and Monocle 3, setting the start clusters as subtypes of lowest differentiation scores and the end clusters regarding diffusion map result and previous conventions^39,41,43,52^.

### Gene set scoring

R package AUCell (v3.18) was used to calculate the score of gene sets for the differentiation and functional analysis^53^. Here, we first ranked the genes from highest to lowest value using the AUCell_buildRankings function. Subsequently, the area under the curve score of each cell was calculated using the AUCell_calcAUC function. Because the dataset was preprocessed before the analysis, the percentage of genes considered was manually modified depending on the number of HVGs. The reference gene sets used for AUCell scoring were chosen based on prior knowledge and research^28,43,54^ (Supplementary Table 4).

We also used scanpy.tl.score_genes under default parameters to score additional gene sets. For cell type identification, markers of neutrophils and macrophages were derived from PanglaoDB^55^ (Supplementary Table 2). For functional analyses related to antigen loading, antigen-processing proteases, antigen processing and presentation, and M1/M2 pathways, we used gene sets from Wu et al.^52^ and Azizi et al.^23^. The top 30 DEGs sorted by ranking within each subtype were used for validation (Supplementary Tables 3 and 5). For cross-species validation, the top 30 mouse orthologous DEGs sorted by ranking were used (Supplementary Table 6). The top 10 DEGs sorted by ranking within *SPP1*^*hi*^ Mφs were scored on all cell types before survival analysis to confirm their specificity.

### Bulk deconvolution

We performed bulk deconvolution using the R package BisqueRNA function ReferenceBasedDecomposition (v1.0.5) to validate cell subtypes and subsequently stratify patients^56^. To compare TNBCs and non-TNBCs, we used TCGA and GSE176078^26^. The results were used to compare the cell type frequencies. To conduct clinical analyses, we retrieved The Cancer Genome Atlas (TCGA) and Molecular Taxonomy of Breast Cancer International Consortium (METABRIC) bulk RNA-seq data followed by selecting TNBCs based on clinical information using cBioPortalData (v2.16.0)^52,57^. To calculate the proportions of each cell type or subtype within each sample, the single-cell count reference matrix and bulk RNA-seq data were used as inputs, while other parameters were set to default.

We initially filtered out samples that lacked mononuclear phagocytes or neutrophils and calculated the correlations among individuals based on subtype composition, followed by hierarchical stratification using R package ComplexHeatmap (v2.20.0)^58^.

To validate our results, we used another bulk deconvolution method, BayesPrism (v2.2.2)^59^. Using the same data as the reference, we deconvoluted TCGA TNBC samples. Next, we grouped patients into high and low SPP1 lineage, using the median value of the combined abundance of *SPP1*^*hi*^ Monos and *SPP1*^*hi*^ Mφs.

### Survival analysis

In this study, survival analyses of patients were performed based on the expression levels of marker genes as well as based on the abundances inferred by bulk deconvolution. In terms of gene expression, we used TCGA bulk RNA-seq data. We first calculated the expression levels of the top 10 DEGs in each subtype by summation. Subsequently, we scored the expression of DEGs in each patient with TNBC of the TCGA cohort. The distribution of these scores across patients was visualized using a histogram, which helped identify a reasonable cutoff point for patient stratification.

To conduct survival analysis in terms of proportion, we initially filtered out samples that lacked the desired subtype followed by categorization using the gene-expression histogram. Because the TCGA data were insufficient for clustering samples based on subtype proportion, we turned to the METABRIC database. The samples were subjected to hierarchical stratification, yielding adequate categorization that corresponded to each subtype.

Subsequently, we plotted Kaplan–Meier survival curves using R packages survival (v3.7.0) and survminer (v0.4.9). Cox regression was used to determine the *P* value along with the hazard ratio and 95% confidence intervals.

### Spatial transcriptomics analysis

A published dataset composed of 43 TNBC Visium samples^60^ was used. We filtered out the low-quality Visium spots that had <2500 or >75,000 counts. Pathway analysis and transcription factor activity inference were performed using decoupleR (v1.7.0)^61^.

### Spatial deconvolution

To map the cell subtypes that we identified in the scRNA-seq analysis at a spatial level, we applied cell2location^62^ as described previously^63,64^. In brief, the negative binominal regression model, which is implemented in cell2location, was applied with the default parameters to the reference scRNA-seq data to estimate the reference signature of the cell subtypes. The calculated reference matrix derived from the combined dataset was used to estimate the spatial abundance of each cell type in each sample. Pathways correlating with macrophage abundance were assessed using Pearson’s *R* correlation. Correlations were regarded as significant if the *P* value of the respective pathway in each spot and the *P* value of the correlation analysis were both <0.05. Moreover, we clustered Visium spots on the basis of the proportion of the inferred cell types.

### CellChat analysis

To infer cell-to-cell interactions, we applied the R package CellChat^65^. Potential ligand–receptor interactions were predicted using the function computeCommunProb. The results were visualized using the function netVisual_chord_gene or netVisual_bubble.

### pySCENIC analysis

To elucidate putative upstream regulators, we used pySCENIC (v0.12.1) across the subtypes and region clusters^53^. In brief, we inferred the co-expression network by using GRNBoost2, followed by regulon prediction. This yielded 57, 103 and 98 regulons in mononuclear phagocytes, neutrophils and region clusters, respectively. The regulon activity per cell or spot was computed using AUCell function. The *z* scores of regulon activity in each subtype or cluster were computed. We then combined *SPP1*^*hi*^ Mφs, *VEGFA*^*hi*^ neutrophils and the angiogenic niche (region cluster 1) to identify overlapping transcription factors.

### Cross-species validation

The mouse-human ortholog table was downloaded from the Mouse Genome Informatics database. After obtaining the mouse orthologs of human myeloid subtype markers, the top 30 genes in murine scRNA-seq datasets were scored as described in the ‘Gene set scoring’ section.

We compared the human myeloid subtypes we detected with the mouse myeloid subtypes in the EMT6 samples as described previously^18^. In brief, we normalized the data and calculated the HVGs in each species. Only genes that were conserved in both species were retained. These were combined to generate a median-normalized average expression count matrix for the subset. After log transformation, hierarchical clustering was performed on the genes and cell subtypes.

### Multiplexed immunofluorescence

A 5-micrometer-thick formalin-fixed, paraffin-embedded tissue section from a patient previously analyzed by scRNA-seq was stained using multiplexed immunofluorescence (mIF). Prior multiplexed staining the staining pattern of each primary antibody was observed by conventional 3,3′-diaminobenzidine (DAB) immunohistochemistry using the Bond Polymer Refine Detection kit (DS9800; Leica). Multiplexed staining was performed after the standard Opal staining protocol. In brief, slides were deparaffinized, rehydrated and permeabilized. After antigen retrieval, slides were incubated with primary antibodies, and Opal dyes. The primary antibodies were as follows: SPP1 (HPA027541; Atlas; 1:300), ADM (TA806874; Origene; 1:2,000), VEGF (sc-7269; Santa Cruz; 1:4,000); CD68 (ab955; abcam; 1:12,000); CD66b (NB1000-77808; Novus; 1:400), Apelin (ab230536; abcam; 1:50). Nuclear counterstain was performed using DAPI. Images were captures using PhenoImager HT (akoya).

### RNA isolation and reverse transcription-quantitative PCR (RT–qPCR)

Total RNA was obtained using TRIzol (15596026; Invitrogen), following the manufacturer’s instructions. Subsequently, cDNA was synthesized utilizing the GoScript Reverse Transcription System (A5000; Promega) according to the manufacturer’s protocol. Quantitative PCR (qPCR) was performed on a QuantStudio 6 Flex Real-Time PCR system (Applied Biosystems) using Power SYBR Green PCR Master Mix (4367659; Applied Biosystems). Sequences of used primers are listed in Supplementary Table 7. Expression levels of target genes were normalized to *RPL37A* or *Actb*. Fold changes were determined by the comparative Ct method (ΔΔCt).

### Immunofluorescence

Cells, cultured on confocal dishes (101350; SPL), were washed twice with 1× PBS. Subsequently, cells were fixed with 2% paraformaldehyde (BPP-9004; T&I) for 15 min. Cells were permeabilized with 0.2% Triton X-100 (TRX01; LPS Solution) in PBS, followed by washing using PBS and blocking in PBS supplemented with 0.1% bovine serum albumin (BSAS 0.1; BovoStar), 2.5% FBS for 1 h. Cells were incubated in blocking solution containing primary antibodies: anti-SPP1 (ab214050; abcam; 1:100) and anti-ADM (TA806874; OriGene). After overnight incubation, cells were washed in PBS and incubated in blocking solution with secondary antibodies: Alexa Fluor 488-conjugated anti-mouse (A28175; Invitrogen; 1:500) and Alexa Fluor 647-conjugated anti-rabbit (A32733; Invitrogen; 1:500). Finally, nuclei were stained with NucBlue Fixed Cell Stain ReadyProbes reagent (R37606; Invitrogen) and mounted with Anti-Fade Fluorescence Mounting Medium (AB104135; abcam). Images were acquired with an LSM800 confocal microscope (Carl Zeiss).

### Western blot

Cells were lysed with lysis buffer (50 mM Tris pH 7.4, 150 mM NaCl, 1 mM EDTA, 1% NP-40 and 0.25% sodium deoxycholate) supplemented with protease inhibitor cocktail. The lysate samples (25 µg per lane) were loaded onto 10–12% sodium dodecyl sulfate polyacrylamide gel and separated by electrophoresis (SDS–PAGE). The proteins were transferred to polyvinylidene fluoride membrane (Bio-Rad Laboratories) by electroblotting. The membranes were blocked with 3% bovine serum albumin-containing blocking buffer (Humost) and incubated with primary antibodies (anti-SPP1; ab214050; abcam) overnight at 4 °C. After three consecutive washing steps with TBS-T solution, the membranes were incubated with HRP-conjugated secondary antibodies for 2 h at room temperature. The protein bands were visualized using enhanced chemiluminescence ECL detection reagent (#32106; Pierce) and imaged with the ChemiDoc MP Imaging System (Bio-Rad Laboratories).

## Results

### Comparison of TNBCs with non-TNBCs highlights the importance of myeloid cells

**Fig. 1:**
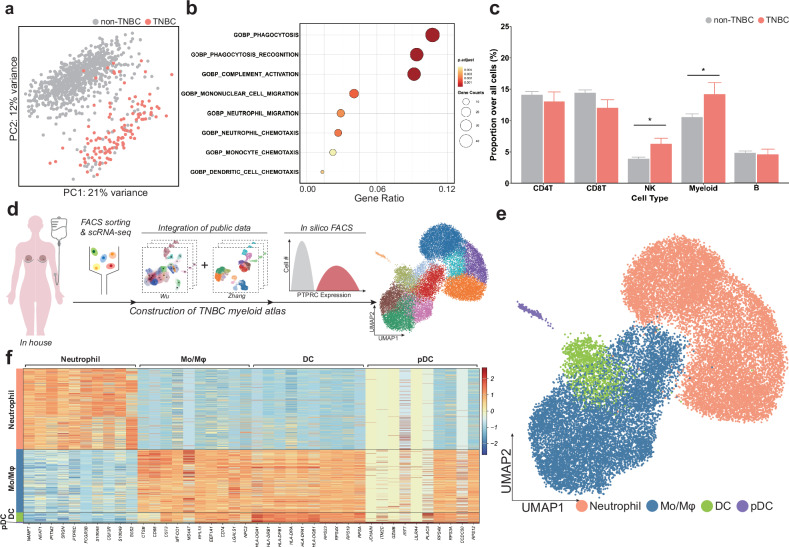
Comparing TNBCs with non-TNBCs highlights the importance of the myeloid population in TNBC. **a** The individual bulk RNA-seq data of the TNBCs and non-TNBCs in the TCGA-BRCA cohort were subjected to PCA. This revealed clear gene expression differences between TNBCs and non-TNBCs. **b** Dot plot showing the GO analysis results of the TNBC DEGs. The genes that were upregulated in TNBCs compared with in non-TNBCs relate to myeloid cell function. **c** Bar plot comparing the abundance of the indicated immune cells in the TCGA-BRCA cohort. The TNBCs demonstrate greater myeloid cell infiltration. **d**–**f** Construction and clustering of our TNBC myeloid cell scRNA-seq atlas: schematic depiction of the experimental workflow used to construct and characterize the myeloid landscape in human TNBC (the scRNA-seq data consist of in-house (*n* = 5) and publicly available samples (GSE176078, *n* = 10; GSE169246, *n* = 10)) (**d**) UMAP visualization of the intratumoral myeloid cells (each cell type was defined on the basis of their expression of canonical markers and PanglaoDB cell-type marker scores; Mo/Mφ, monocytes and macrophages) (**e**) heatmap showing the expression pattern of the top 10 DEGs in the different myeloid cell types (**f**).

Unlike non-TNBC breast cancers, TNBC lacks effective treatments and is associated with a poor prognosis, as it does not exhibit targetable gene expression or amplification. Therefore, to identify potential therapeutic targets specific to TNBC, we investigated whether the TME profile of TNBC differs significantly from that of non-TNBC. To this end, we compared 116 TNBCs with 875 non-TNBCs using their bulk RNA-seq results from the TCGA breast invasive carcinoma (TCGA-BRCA) database. Indeed, PCA showed not only that the TNBCs differed strongly from the non-TNBCs (Fig. 1a), but also that the first two principal components included multiple genes that related to myeloid function (Supplementary Fig. 1a, b). Moreover, when the DEGs between the TNBCs and non-TNBCs were subjected to GO analysis, TNBCs demonstrated enrichment of myeloid-related pathways (Fig. 1b). Thus, strong myeloid cell involvement clearly distinguishes TNBCs from non-TNBCs.

To assess this further, we compared the TNBCs and non-TNBCs in the TCGA-BRCA database in terms of myeloid cell abundance. For this, the TCGA-BRCA cohort was deconvoluted with BisqueRNA using scRNA-seq data from a study encompassing both TNBCs and non-TNBCs as a reference^26^. This showed that myeloid cells, compared with other immune cell types, were more significantly abundant in TNBCs (Fig. 1c). These observations, together with the fact that myeloid cells predict worse prognosis in breast cancer^66,67^, led us to focus our attention on the myeloid population in TNBCs.

### Investigation of the myeloid landscape in TNBC reveals four major cell types

We thus aimed to elucidate the myeloid landscape inside TNBCs and decipher their functions within the TME using scRNA-seq (Fig. 1d). To comprehensively delineate the myeloid environment in TNBC, we isolated myeloid cells from patient TNBC samples that were obtained via surgical resection and subjected the cells to scRNA-seq (Fig. 1d, Supplementary Fig. 1c and Supplementary Table 1). We also further expanded our dataset using batch balanced *k*-nearest neighbors (BBKNN) to integrate the myeloid cells from the publicly available TNBC datasets of Wu et al.^26^ and Zhang et al.^34^ (Fig. 1d). Cell annotation based on canonical markers and PanglaoDB marker gene scores (Supplementary Fig. 1d, e and Supplementary Table 2) led to a single-cell atlas that contained 24,217 high-quality myeloid cells. UMAPs and heatmaps of the annotated database showed that our atlas primarily consisted of four myeloid cell types: monocytes/macrophages, neutrophils, DCs and pDCs (Fig. 1e, f).

### Intratumoral neutrophil analysis reveals heterogeneity in development and function

**Fig. 2:**
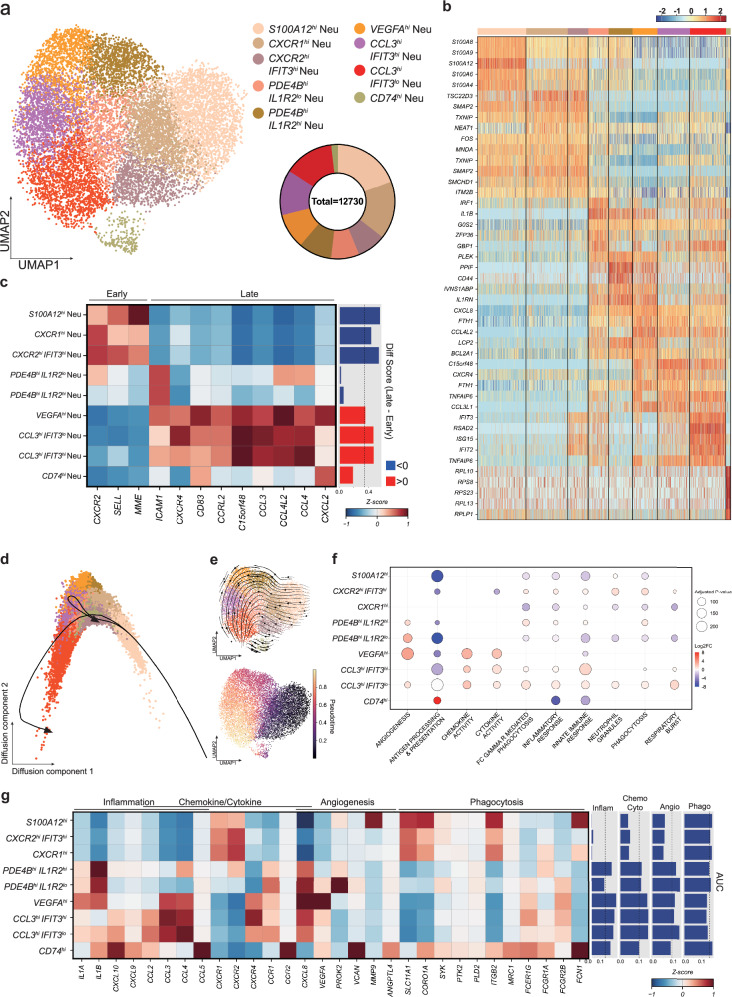
Intratumoral neutrophil subtypes display heterogeneity in lineages and functions. **a** UMAP of the nine neutrophil subtypes and a pie chart depicting the proportions of each subtype. **b** Heatmap showing the expression pattern of the DEGs across the neutrophil subtypes. **c** Matrix plot presenting the expression level of genes that relate to the maturation state of the neutrophils. The bar plot indicates the maturation score, which was calculated by subtracting the early maturation score from the late maturation score. Each score was calculated by using AUCell. The dashed line marks the mean maturation score of all neutrophil subtypes. **d** Low-dimensional reduction plot of neutrophils using a diffusion-map approach. The arrows indicate the trajectories that were calculated by slingshot. The trajectory starts from the *S100A12*^*hi*^ neutrophils and ends with the *CCL3*^*hi*^ *IFIT3*^*lo*^ neutrophils and *CD74*^*hi*^ neutrophils. **e** The differentiation of neutrophils was also estimated by using scTour. These data are displayed in a vector map (top) and as pseudotime scores (bottom). These data show that the *S100A12*^*hi*^ neutrophils differentiate continuously into *CCL3*^*hi*^ *IFIT3*^*hi*^ neutrophils. **f** The pathway activities in the neutrophil subtypes were calculated with GSVA. The color and size of each dot correspond to log_2_ fold change and adjusted *P* value, respectively. **g** The gene expression in the neutrophil subtypes of functions that have been detected previously in tumor-associated neutrophils. The bar plot indicates the score of the corresponding gene set, which was calculated using AUCell. The dashed lines mark the mean score of the neutrophil subtypes.

The neutrophils and monocytes/macrophages were then characterized by subclustering and in-depth analysis of each subtype. Nine intratumoral neutrophil subtypes were detected (Fig. 2a, b, Supplementary Fig. 2a, b and Supplementary Table 3). Because neutrophils cannot proliferate further, their heterogeneous phenotypes in the TNBCs must be driven by reprogramming of the cells once they infiltrate the solid tumor^41,68^. To elucidate this reprogramming process, the trajectories of the different neutrophil subtypes were determined. First, we found that none of the neutrophil subsets expressed neutrophil precursor markers, confirming their maturation upon tumor infiltration, consistent with previous findings^69^ (Supplementary Fig. 2c). To determine how neutrophils were reprogrammed after tumor entry, we categorized the subtypes into early or late stages of differentiation. Genes previously identified to mark the neutrophil maturation process in physiological or inflammatory settings are insufficient to capture the tumor context, emphasizing the need for additional markers^41,70^. Therefore, we defined early-stage neutrophils based on genes upregulated in circulation or normal physiology^69,70^. By contrast, late-stage neutrophils were defined by genes upregulated in intratumoral neutrophils^43^ (Supplementary Table 4). To obtain a differentiation score reflecting the developmental stage of the neutrophil subtypes, the AUC scores of the early/late marker-gene groups were calculated and the early score was subtracted from the late score (Fig. 2c). This analysis showed that the *S100A12*^*hi*^, *CXCR1*^*hi*^ and *CXCR2*^*hi*^ *IFIT3*^*hi*^ neutrophils were at an early stage, while the *VEGFA*^*hi*^, *CCL3*^*hi*^ *IFIT3*^*lo*^, *CCL3*^*hi*^ *IFIT3*^*hi*^ and *CD74*^*hi*^ neutrophils belonged to a late stage (Fig. 2c).

Next, we aimed to further characterize the developmental trajectory of the intratumoral neutrophils. We first used a diffusion-map approach to order the cells on the basis of transitional probabilities^41,71^ (Fig. 2d and Supplementary Fig. 2d). The *S100A12*^*hi*^ neutrophils, which were early-stage neutrophils (Fig. 2c), were located at one end of the diffusion map (Fig. 2d). This is consistent with their high expression of *MMP9* and *SELL* (Supplementary Fig. 2e), which are genes that function during neutrophil migration and infiltration^52^. Analysis of the diffusion map with slingshot showed that the trajectory from the *S100A12*^*hi*^ neutrophils diverged into the late-stage *CCL3*^*hi*^ *IFIT3*^*lo*^ and *CD74*^*hi*^ neutrophils (Fig. 2d). Other trajectory algorithms scTour and Monocle 3 revealed similar patterns (Fig. 2e and Supplementary Fig. 2f). Meanwhile, *CXCR2* and *CXCR4* expression, often used to distinguish young and aged neutrophils, respectively^72^, were consistent with the results, although late-phase *CD74*^*hi*^ neutrophils only expressed low levels of *CXCR4* (Fig. 2c). This may be due to their different origin suggested in a recent study^73^.

Neutrophils play various roles in solid tumors including inflammation, chemokine/cytokine signaling, angiogenesis and phagocytosis^28^. To determine the functions of the nine neutrophil subtypes, we used GSVA (Fig. 2f) and the expression patterns of related genes (Fig. 2g and Supplementary Table 4). The three early-stage neutrophil subtypes displayed higher phagocytic activities and enrichment in neutrophil granule-related pathways. The late-stage *VEGFA*^*hi*^, *CCL3*^*hi*^ *IFIT3*^*hi*^ and *CCL3*^*hi*^ *IFIT3*^*lo*^ neutrophils demonstrated enriched chemokine, cytokine and inflammation activity. Notably, *VEGFA*^*hi*^ neutrophils had enriched levels of angiogenesis, which is consistent with their high expression of vascular endothelial growth factor A (VEGFA) (Fig. 2f, g).

The relatively rare *CD74*^*hi*^ neutrophil subtype displayed a unique phenotype. Even though this subtype was classified as a late-stage neutrophil subtype based on trajectorial analyses, it displayed lower levels of chemokine, cytokine, inflammatory and angiogenic functions, unlike other late subtypes. Instead, this subtype displayed enhanced antigen processing and presentation activity (Fig. 2f, g). Indeed, upon analyzing the expression pattern of antigen-presenting neutrophil markers and antigen-processing- and antigen-presentation related genes^74^, we observed high expression levels in *CD74*^*hi*^ neutrophils but not in other subtypes (Supplementary Fig. 3a, b). We further verified the antigen-presentation capability of *CD74*^*hi*^ neutrophils by GSEA (Supplementary Fig. 3c). Because neutrophils have not been classified as professional antigen-presenting cells, we asked how well they presented antigen compared with tumoral DCs. We compared *CD74*^*hi*^ neutrophils with the five DC subtypes identified in this study (Supplementary Fig. 3d) and found lower antigen-presenting score in *CD74*^*hi*^ neutrophils in TNBC (Supplementary Fig. 3e).

Some of our in-house TNBC neutrophil subtypes resembled the neutrophil subtypes detected in pan-cancer neutrophils atlas of Wu et al.^52^. Indeed, comparison of the gene signatures showed that the *CD74*^*hi*^ neutrophils correlated very closely with their HLA-DR^+^ CD74^+^ neutrophil subset. Other subtypes showed partial overlap, with some subtypes from each dataset being subsumable or groupable under broader clusters in the other dataset, reflecting a complex yet incomplete correspondence between them (Supplementary Fig. 3f).

### Intratumoral monocytes and macrophages exhibit high heterogeneity and diverse origins

**Fig. 3:**
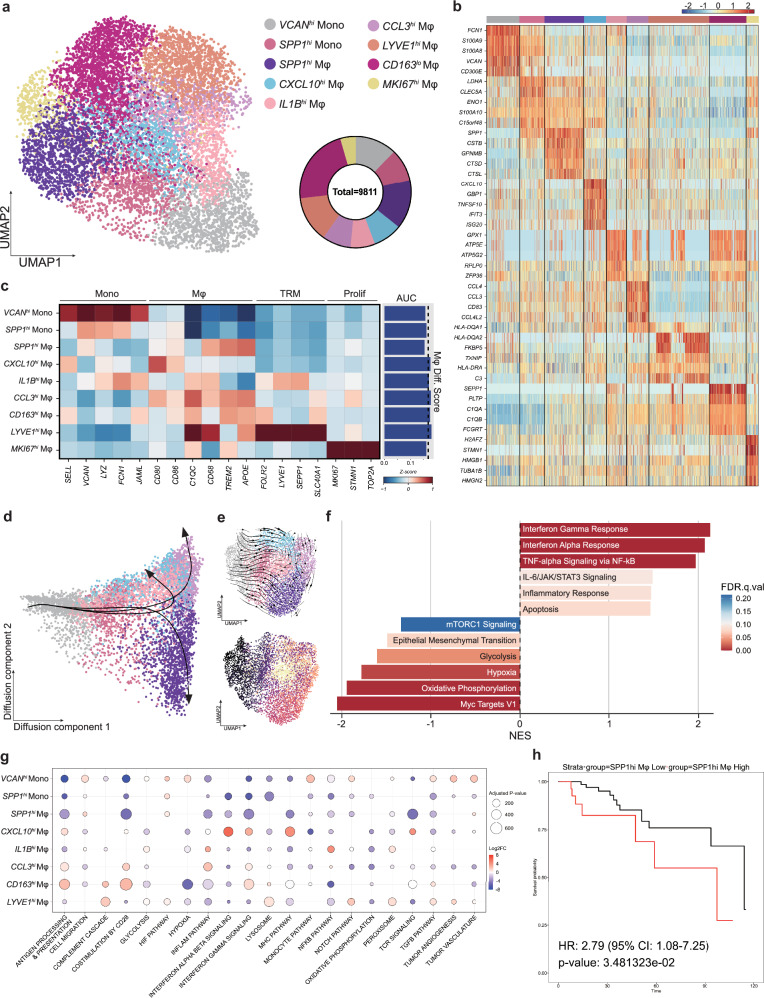
Monocytes and macrophages in TNBCs display distinct lineages that are respectively characterized by inflammation and metabolic stress. **a** UMAP of the monocytes and macrophages in TNBCs. The pie chart presents the proportions of each subtype. **b** Heatmap depicting the expression of the DEGs across the monocyte and macrophage subtypes. **c** Expression in all subtypes of the genes that mark each subtype. The bar plot displays the macrophage differentiation score (Mφ Diff), which was computed using AUCell on the basis of the gene set GO: 0030225. The dashed line indicates the mean AUC score of the subtypes. The monocyte subtypes had lower differentiation scores, whereas all macrophage subtypes apart from *SPP1*^*hi*^ Mφs and *MKI67*^*hi*^ Mφs had scores above the mean. **d** Two-dimensional visualization of the differentiation trajectories of the monocytes and monocyte-derived macrophages, as determined with a diffusion-map approach. The arrows indicate the trajectories that were calculated with slingshot. They all start from *VCAN*^*hi*^ Monos but end with either *SPP1*^*hi*^ Mφs, *IL1B*^*hi*^ Mφs or *CCL3*^*hi*^ Mφs. **e** Estimated differentiation trajectory of monocytes and monocyte-derived macrophages, as determined with scTour. These data are displayed in a vector map (top) and as pseudotime scores (bottom). The lineage that results in *SPP1*^*hi*^ Mφs is distinct from the classical lineage that involves the differentiation of *IL1B*^*hi*^ Mφs into *CCL3*^*hi*^ Mφs. **f** GSEA comparing the classical lineage and SPP1 lineage. The classical lineage is marked by enhanced inflammation-related pathways while the SPP1 lineage displays increased metabolic stress-related pathway activity. **g** The GSVA-calculated pathway activities in the monocyte/macrophage subtypes. The color and size of each dot indicate the log_2_ fold change and adjusted *P* value, respectively. SPP1hi Mφs are enriched in glycolysis, hypoxia and tumor vasculature pathways. **h** Kaplan–Meier plot depicting the OS of patients with TNBC in the TCGA cohort after their stratification according to their high (red) or low (black) expression of *SPP1*^*hi*^ Mφ markers. The *P* value was calculated using Cox regression. HR, hazard ratio; CI, confidence interval.

Based on the expression pattern of canonical markers, we identified two monocyte and seven macrophage (Mφ) subtypes (Fig. 3a, b, Supplementary Fig. 4a and Supplementary Table 5). Compared with the monocyte subtypes, the macrophage subtypes had higher AUC scores in the GO geneset ‘macrophage differentiation’ (GO: 0030225, Mφ Diff) except *SPP1*^*hi*^ Mφs, suggesting that this population diverges apart from physiological or inflammatory context in which the GO geneset was curated (Fig. 3c and Supplementary Table 4).

Macrophages can be classified based on their origin into monocyte-derived macrophages and tissue-resident macrophages (TRMs)^25,42^. We found the *LYVE1*^*hi*^ Mφs expressed high levels of TRM-marker genes^8,42^ (Fig. 3c and Supplementary Fig. 4b). The *CD163*^*hi*^ Mφs have been identified as TRMs previously^25^.

We next assessed the trajectories to decipher the development from monocytes into TAMs. Given their origin, the *LYVE1*^*hi*^ Mφ and *CD163*^*hi*^ Mφ TRMs were excluded from this analysis. The *MKI67*^*hi*^ Mφs were also excluded because they were a proliferative subtype derived from multiple subtypes, including both monocyte-derived macrophages and TRMs. *VCAN*^*hi*^ monocytes, which had low macrophage differentiation scores (Fig. 3c), were positioned at one end of the diffusion map, indicating them as the starting point of the trajectory (Fig. 3d and Supplementary Fig. 4c). Also, slingshot revealed three lineages that ended with *SPP1*^*hi*^ Mφs, *CXCL10*^*hi*^ Mφs and *CCL3*^*hi*^ Mφs, respectively (Fig. 3d). However, further analysis using scTour and Monocle 3 showed that the latter two trajectories are connected. Thus, we could construct two distinct monocyte/macrophage lineages: the classical lineage, where *VCAN*^*hi*^ monocytes subsequently differentiate into *IL1B*^*hi*^ Mφs, *CXCL10*^*hi*^ Mφs and *CCL3*^*hi*^ Mφs, and the SPP1 lineage, in which *VCAN*^*hi*^ monocytes differentiate into *SPP1*^*hi*^ monocytes and then *SPP1*^*hi*^ Mφs (Fig. 3e and Supplementary Fig. 4d). The two lineages were also unique in terms of function (Fig. 3f). GSEA showed that the classical lineage was enriched in inflammation-related pathways while the SPP1 lineage was enriched in metabolic stress-related pathways such as hypoxia and fatty-acid metabolism.

We also analyzed the enriched pathways in each subtype by using GSVA. This showed that the macrophages in the TME of TNBC play diverse roles (Fig. 3g and Supplementary Table 4). The *CXCL10*^*hi*^ Mφs and *CCL3*^*hi*^ Mφs were enriched in inflammation-related pathways (inflammation and interferon signaling); the *CCL3*^*hi*^ Mφs also displayed enhanced antigen processing and presentation activity along with the *CD163*^*hi*^ Mφs, and *SPP1*^*hi*^ Mφs showed downregulation of all of these pathways and instead exhibited upregulation of the HIF pathway. Interestingly, *SPP1*^*hi*^ Mφs also demonstrated upregulation of the tumor vasculature pathway, suggesting an angiogenic potential in response to metabolic stress, specifically hypoxia (Fig. 3g).

Tumor macrophages have been classified as antitumoral M1 and protumoral M2 subtypes^75^. However, we observed that some macrophage subtypes co-expressed M1 and M2 signature genes (Supplementary Fig. 4e). This supports studies that suggest a macrophage spectrum of diversity and plasticity contrary to the conventional dichotomous M1/M2 classification^22,23^.

We next compared our TNBC monocyte/macrophage subtypes with those found in normal breast tissue by retrieving scRNA-seq data from a previous publication^35^ (Supplementary Fig. 4f). We found an unexpectedly strong correlation between the normal-tissue subtypes and the TNBC subtypes (Supplementary Fig. 4g). In fact, even protumoral *SPP1*^*hi*^ Mφs correlated strongly with Mφ1 and Mφ2 in normal breast tissue. We hypothesized that, despite these high correlations, the *SPP1*^*hi*^ Mφs and normal-tissue Mφ1 and Mφ2 subtypes may differ in terms of functional capabilities. To test this, we compared the scores of the enriched pathways in *SPP1*^*hi*^ Mφs to Mφ1, and Mφ2. Indeed, the intratumoral *SPP1*^*hi*^ Mφs had a higher tumor vasculature score than the normal-tissue Mφ1 and Mφ2 (Supplementary Fig. 4h). Thus, *SPP1*^*hi*^ macrophages may adopt heightened angiogenic functions in the context of the TME.

To assess the protumoral functions and clinical impact of the *SPP1*^*hi*^ Mφs, we compared the effect of high and low *SPP1*^*hi*^ Mφ signature scores on the survival of patients with TNBC in the TCGA cohort. Strikingly, patients with high *SPP1*^*hi*^ Mφ scores showed worse overall survival (OS) than the patients with low scores (Fig. 3h and Supplementary Fig. 4i–k). Collectively, these results indicate that *SPP1*^*hi*^ Mφs are a stressed macrophage population in TNBC that displays enhanced activity of hypoxia-related pathways and exerts protumoral functions such as angiogenesis, leading to worse prognosis.

### *SPP1*^*hi*^ Mφs localize in hypoxic niches and actively interact with surrounding cancer cells

**Fig. 4:**
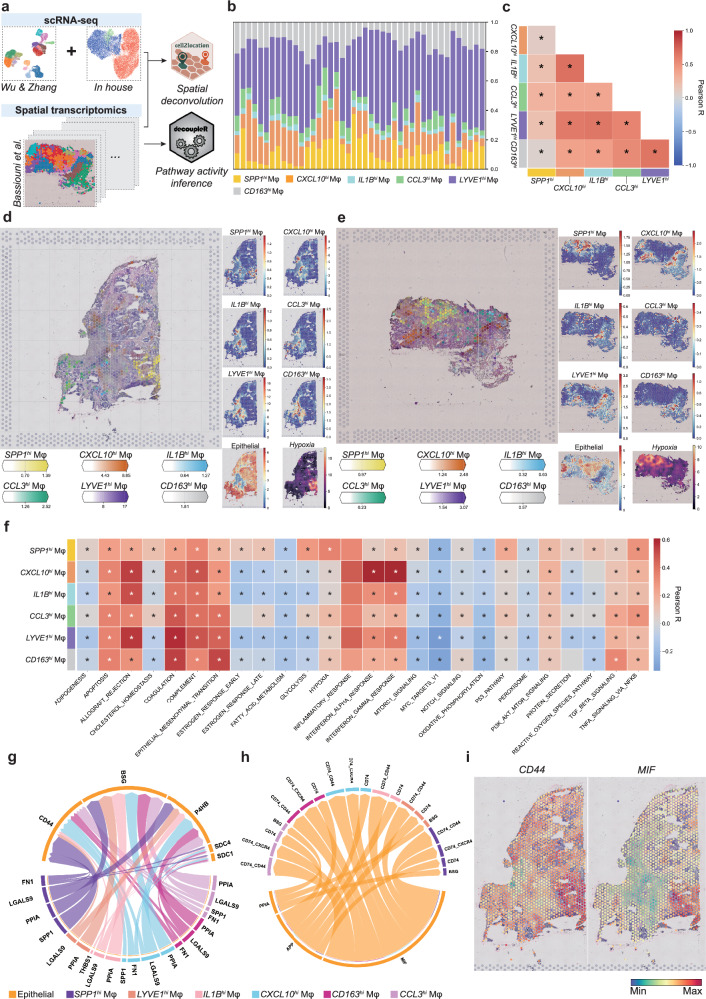
*SPP1*^*hi*^ Mφs colocalize with cancer cells in hypoxic niches, while the other macrophage subsets localize at blood vessels. **a** A schematic depiction of the spatial transcriptomic analysis performed in the present study. Our TNBC myeloid cell scRNA-seq atlas was combined with the publicly available Visium data on TNBC samples (GSE210616)^60^. **b** The estimated proportion of each macrophage subtype in the total macrophage population of each Visium sample. Abundance was calculated using cell2location. **c** Correlation between the macrophage subtype proportions across the Visium spots. *SPP1*^*hi*^ Mφs correlated poorly with the other subtypes. **d**, **e** The spatial distribution of the macrophage subtypes and epithelial cancer cells in the representative Visium slides denoted 094D (**d**) and 395D (**e**). The hypoxia-pathway activity in each Visium spot was also calculated using decoupler. *SPP1*^*hi*^ Mφs colocalized with epithelial cells in hypoxic regions. **f** Correlation between the transcriptomic signatures of various pathways from the MSigDB database and the macrophage subtype proportions across Visium spots. *SPP1*^*hi*^ Mφs show heightened hypoxia-pathway activity. **g** Cell–cell interaction from macrophage subtypes to epithelial cells, as inferred by CellChat analysis. **h** Cell–cell interaction from epithelial cells to macrophage subtypes, as inferred by CellChat analysis. **i** Spatial-expression patterns of the epithelial-ligand MIF and its CD44 receptor on SPP1^hi^ Mφs in 094D, a representative slide. High expression of *CD44* and *MIF* was observed in the hypoxic niches. *P < 0.05.

One limitation of scRNA-seq is that it does not provide information on the spatial arrangement of cells in tissues. As proximity to other cells can considerably impact cell functions^63^, we examined the spatial arrangements of the myeloid cells in TNBC sample sections. For this, we retrieved publicly available 10x Visium data of 43 TNBC sections^60^. We captured spatial localization of myeloid subtypes using cell2location for spatial deconvolution and performed decoupleR pathway enrichment analysis across Visium spots (Fig. 4a).

We observed that the macrophage subtype proportions varied across sections and between patients (Fig. 4b). Spatial correlation analysis of the macrophage subtypes showed that the classical-lineage macrophage subtypes (*CXCL10*^*hi*^ Mφs, *IL1B*^*hi*^ Mφs and *CCL3*^*hi*^ Mφs) and the TRMs (*LYVE1*^*hi*^ Mφs and *CD163*^*hi*^ Mφs) correlated closely with each other (Fig. 4c). By contrast, the *SPP1*^*hi*^ Mφ showed lower correlation with both, the classical-lineage macrophage subtypes and the TRMs (Fig. 4c). Thus, *SPP1*^*hi*^ Mφ may display a unique and isolated spatial arrangement. Notably, we found that *SPP1*^*hi*^ Mφs localized in the hypoxic regions, characterized by high numbers of epithelial cancer cells (Fig. 4d, e).

To identify functions that are enriched with the localization of each subtype, we assessed the correlation between macrophage subtype abundance and the gene signatures of various pathways (Fig. 4f). Consistent with the aforementioned result indicating colocalization of the majority of macrophage subtypes, all macrophage subtypes except *SPP1*^*hi*^ Mφs showed correlation with similar pathway signatures, while *SPP1*^*hi*^ Mφ abundance correlated more strongly with hypoxia and metabolic stress-related signatures (for example, adipogenesis and cholesterol homeostasis).

Given that *SPP1*^*hi*^ Mφs colocalized with cancer cells (Fig. 4d, e), we expected strong interactions between these cell types. To assess the ligand–receptor interactions between the macrophage subtypes and cancer cells, we used CellChat^65^ (Fig. 4g, h). This revealed strong interactions between *SPP1*^*hi*^ Mφs and cancer cells, both acting as sources and targets for each other. Notably, the expression of *MIF* and *CD44*—the ligand and receptor involved in the strongest predicted interactions—was more enriched in hypoxic niches (Fig. 4i and Supplementary Fig. 5a). Overall, the spatial transcriptomics showed that *SPP1*^*hi*^ Mφs and cancer cells colocalize to form a hypoxic niche and probably interact closely.

Meanwhile, the other five macrophage subsets (that is, the classical lineage macrophages *CXCL10*^*hi*^ Mφs, *IL1B*^*hi*^ Mφs and *CCL3*^*hi*^ Mφs and the TRMs *LYVE1*^*hi*^ Mφs and *CD163*^*hi*^ Mφs) colocalized with each other, in proximity to ECs and pericytes (Supplementary Fig. 5b, c), indicating that they are localized near blood vessels. As Zhao et al. suggested that *LYVE1* is an alternative marker of TIM4^+^ perivascular macrophages^76^, we speculated that our *LYVE1*^*hi*^ Mφs correspond to this TRM subtype. This is also supported by their high expression of TRM markers (Fig. 3c and Supplementary Fig. 4b). A recent study on mammary adenocarcinoma showed that perivascular LYVE1^+^ TAMs secrete the platelet-derived growth factor-C (PDGF-C) homodimer, PDGF-CC, which signals to PDGF receptor-α (PDGFRA)-expressing stromal cells involved in blood vessel formation. This signaling promotes the creation of a proangiogenic perivascular niche^77^. This interaction was also evident in our data: both *PDGFC* and *PDGFRA* were expressed near *LYVE1*^*hi*^ Mφs in multiple slides (Supplementary Fig. 5d, e).

### *SPP1*^*hi*^ Mφs colocalize with *APLN*^*hi*^ tip cells and *VEGFA*^*hi*^ neutrophils, thus forming an angiogenic niche

**Fig. 5:**
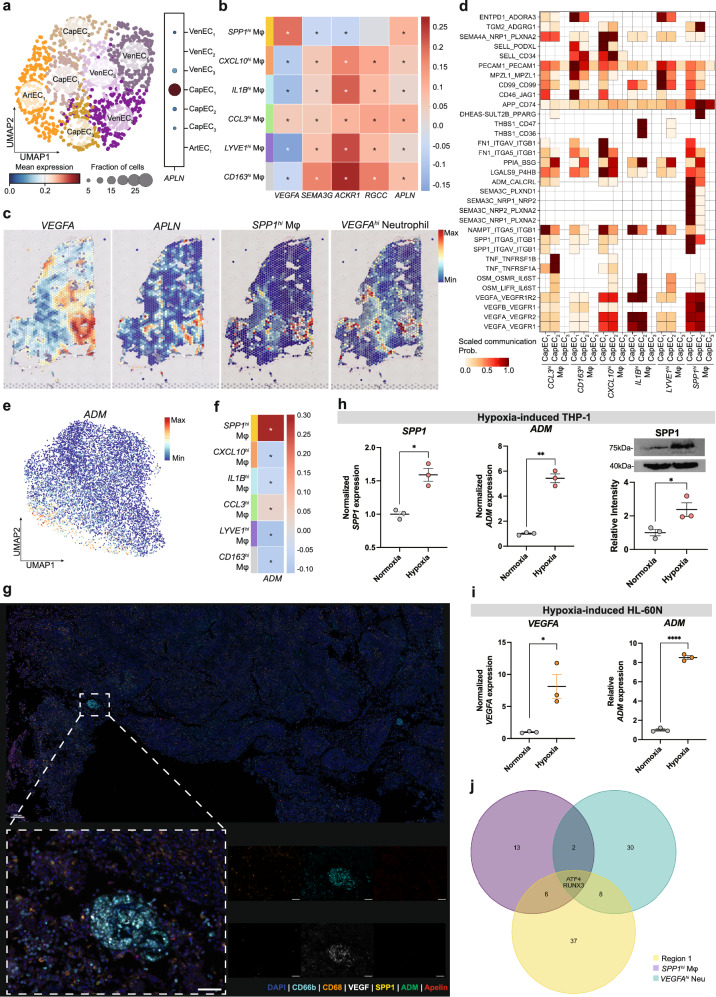
*SPP1*^*hi*^ Mφs reside at hypoxic niches with *VEGFA*^*hi*^ neutrophils and *APLN*^*hi*^ tip cells and promote angiogenesis. **a** UMAP of the intratumoral EC subtypes and their *APLN* expression. Dot size and color of the dotplot correspond to mean expression and fraction of cells, respectively. *APLN* was only expressed by the CapEC_1_ subset. **b** A matrix plot displaying the correlation between the macrophage subtypes and EC markers. SPP1hi Mφs show negative or no correlation with *SEMA3G*, *ACKR1* and *RGCC* but correlate strongly with *VEGFA* and *APLN*. **c** A representative slide (094D) showing the expression of *VEGFA* and *APLN* and the distribution of *SPP1*^*hi*^ Mφs and *VEGFA*^*hi*^ neutrophils. The *SPP1*^*hi*^ Mφs and *VEGFA*^*hi*^ neutrophils colocalized in the region that bears high *VEGFA* and *APLN* expression. **d** A matrix plot depicting the major ligand–receptor pairs between the macrophage subtypes and the three capillary EC subtypes. Of the macrophage subtypes, *SPP1*^*hi*^ Mφs interact most strongly with CapEC_1_. **e** *ADM* expression in the monocytes and macrophages in our atlas. *ADM* expression localizes to the *SPP1*^*hi*^ monocytes and the *SPP1*^*hi*^ Mφs. **f** Spatial correlation between macrophage subtypes and *ADM* expression, as shown by a matrix plot. **g** mIF results showing the presence of an angiogenic niche a patient sample. The colocalization of *SPP1*^hi^ Mφs and *VEGFA*^hi^ neutrophils with APLN-expressing cells can be seen. Scale bars, 200 µm (top) and 50 µm (bottom). **h** Hypoxia induces THP-1 cells into *SPP1*^hi^ Mφ-like cells. PMA-treated THP-1 cells were cultured in hypoxic conditions for 24 h. Expression of *SPP1* and *ADM* was validated by RT–qPCR. The SPP1 protein level was measured by western blot. **i** Hypoxia induces HL-60N cells into *VEGFA*^hi^ neutrophil-like cells. HL-60N cells were exposed to hypoxia, and the expression levels of *VEGFA* and *ADM* were measured by RT–qPCR. **j** A Venn diagram of the significant transcription factors in *SPP1*^*hi*^ Mφs, *VEGFA*^*hi*^ neutrophils and region cluster 1 that were identified by pySCENIC. ATF4 and RUNX3 are activated in all three.

*APLN-*positive endothelial tip cells have been reported to be a capillary EC subtype that plays key roles in initiating angiogenesis^78^. Because *SPP1*^*hi*^ Mφs reside at hypoxic niches (Fig. 4d, e) and were enriched in tumor-vasculature-related pathways compared with *SPP1*^*hi*^-like Mφs in normal breast or the classical-lineage macrophages in TNBC (Fig. 3g and Supplementary Fig. 4h), we speculated that *SPP1*^*hi*^ Mφs interact with angiogenic *APLN*^*hi*^ tip cells. We first verified that *APLN* expression is limited to a small fraction of the capillary ECs in TNBCs (designated CapEC_1_) (Fig. 5a and Supplementary Fig. 6a, b). This allowed us to use *APLN* as a specific angiogenic tip cell marker. Examination of the spatial correlation between the macrophage subtypes and *APLN* expression (Fig. 5b) showed that all macrophage subtypes except *SPP1*^*hi*^ Mφs showed strong spatial correlation with the arterial EC marker *SEMA3G*, the venous EC marker *ACKR1* and the capillary EC marker *RGCC* (Fig. 5b), suggesting their localization in proximity to preexisting blood vessels. By contrast, *SPP1*^*hi*^ Mφs showed negative correlation with these markers, but positive correlation with *APLN* expression (Fig. 5b). This shows that *SPP1*^*hi*^ Mφs do not reside along already established vessel beds, but at sites of early angiogenesis. Interestingly, the niches bearing *SPP1*^*hi*^ Mφs and *APLN*^*hi*^ tip cells showed high levels of *VEGFA* and were also occupied by *VEGFA*^*hi*^ neutrophils (Fig. 5b, c). Furthermore, *SPP1*^*hi*^ monocytes and *PDE4B*^*hi*^*IL1R2*^*hi*^ neutrophils, which are the precursors of *SPP1*^*hi*^ Mφs and *VEGFA*^*hi*^ neutrophils respectively, resided in close proximity to these niches (Supplementary Fig. 6c, d). The colocalization of *SPP1*^*hi*^ Mφs and *VEGFA*^*hi*^ neutrophils with *APLN*^*hi*^ tip cells suggests both interact strongly with the tip cells. To test this, we investigated the cell-to-cell interactions of the capillary ECs with the myeloid subtypes (Fig. 5d and Supplementary Fig. 6e). The *SPP1*^*hi*^ Mφs and *VEGFA*^*hi*^ neutrophils both interacted strongly with the capillary ECs, especially the CapEC_1_ subset, which represents the *APLN*^*hi*^ tip cell. The interaction between adrenomedullin (ADM) and calcitonin receptor-like receptor (CALCRL) was especially strong between the *SPP1*^*hi*^ Mφs and CapEC_1_s (Fig. 5d). ADM is a vasodilating peptide hormone that was recently found to be secreted by hypoxic macrophages and to destabilize tumor blood vessels in glioblastoma^79^. We observed that ADM was exclusively expressed in the protumoral SPP1-lineage cells, namely *SPP1*^*hi*^ monocytes and *SPP1*^*hi*^ Mφs (Fig. 5e). Moreover, the *APLN*^*hi*^ tip cells highly expressed the CALCRL receptor and the RAMP2 and RAMP3 coreceptors, which all participate in ADM signaling^80^ (Supplementary Fig. 6f). These results are supported by spatial correlation between *SPP1*^*hi*^ Mφs and *ADM* expression: the Visium spots with high *SPP1*^*hi*^ Mφ abundance expressed higher levels of *ADM* (Fig. 5f and Supplementary Fig. 6g). With regard to the interactions between *VEGFA*^*hi*^ neutrophils and *APLN*^*hi*^ tip cells, we found strong VEGF signaling, which was proposed as an inducer of *APLN*^*hi*^ tip cell development (Supplementary Fig. 6e). Notably, ADM expression was also present in *PDE4B*^hi^ *IL1R2*^lo^ neutrophils which are the precursors of *VEGFA*^hi^ neutrophils, indicating that neutrophils may engage in ADM signaling (Supplementary Fig. 6h). Collectively, these results show that *SPP1*^*hi*^ Mφs and *VEGFA*^*hi*^ neutrophils colocalize with *APLN*^*hi*^ tip cells to form an angiogenic niche, and that these myeloid subtypes actively engage in modulating the tumor vasculature by expressing ADM and VEGFA. As our spatial analyses were focused at the transcriptome level, we further validated our results at the protein level. mIF effectively revealed the presence of the angiogenic niche (Fig. 5g).

We conducted in vitro experiments to model the development of myeloid cells within hypoxic environments. THP-1 cells differentiated with PMA were cultured under hypoxic conditions, resulting in the upregulation of SPP1 and ADM expression (Fig. 5h and Supplementary Fig. 6i). Similarly, HL-60 cells differentiated into neutrophil-like cells (HL-60N) using DMSO exhibited increased expression of VEGFA and ADM in response to hypoxia, thereby recapitulating our computational observations in vitro (Fig. 5i). These findings were further validated using BMDMs (Supplementary Fig. 6j).

Given that the precursors of *SPP1*^*hi*^ Mφs and *VEGFA*^*hi*^ neutrophils resided at similar locations and shared proangiogenic functions despite their different ontologies, we hypothesized that the local hypoxic/angiogenic environment activates the same transcription factor in both cell types and that this then induces the proangiogenic reprogramming and function of these myeloid subtypes in situ. To test this, we used pySCENIC to assess the regulon activities in the monocytes/macrophages and neutrophils (Supplementary Fig. 7a–c). We then clustered the Visium spots of all slides according to their cell-type proportion as inferred with cell2location, which revealed 12 region clusters (Supplementary Fig. 7d). Region cluster 1 expressed high levels of *VEGFA* and *APLN* and was an angiogenic niche that was abundantly endowed with *SPP1*^*hi*^ Mφs and *VEGFA*^*hi*^ neutrophils (Supplementary Fig. 7e, f). When we examined the regulon activities of the *SPP1*^*hi*^ Mφs, *VEGFA*^*hi*^ neutrophils and region cluster 1, two common transcription factors were identified: ATF4 and RUNX3 (Fig. 5j). ATF4 is induced in macrophages by metabolic stress^81^ and has been reported to be a critical transcription factor for *VEGFA* expression by alveolar bone macrophages^82^. We also found that ATF4 regulon activity was higher and more specific than RUNX3 regulon activity (Supplementary Fig. 7g, h). Furthermore, analysis of public chromatin immunoprecipitation followed by sequencing (ChIP–seq) data revealed that the transcription factor ATF4 is upstream of *VEGFA*, *ADM* and *SPP1* (Supplementary Fig. 7i). Collectively, these results suggest that ATF4 is induced in *SPP1*^*hi*^ Mφs and *VEGFA*^*hi*^ neutrophils by metabolic stress, including hypoxia, which in turn generates the potent angiogenic features thereby promoting *APLN*^*hi*^ tip cell activity.

### Bulk deconvolution validates myeloid subtypes in clinical datasets and enables stratification that reveals the clinical significance of these subtypes

**Fig. 6:**
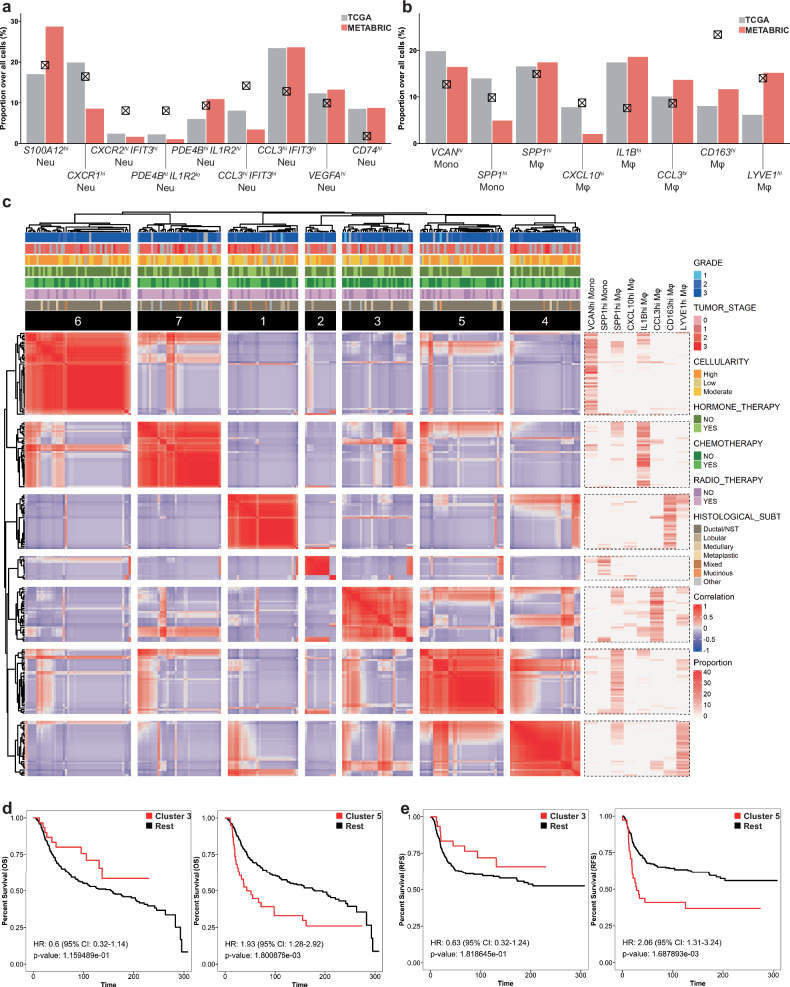
Bulk deconvolution stratifies patients on the basis of myeloid subtype proportions and shows that SPP1^hi^ Mφs have clinical protumoral activities. **a**, **b** Validation and quantification of our neutrophil subtypes (**a**) and monocyte/macrophage subtypes (**b**) in the TCGA and METABRIC cohorts. The X-bearing boxes indicate the proportion of these subtypes in our scRNA-seq atlas. **c** Hierarchical clustering of the TNBC patients in the METABRIC cohort on the basis of correlations between the monocyte/macrophage subtype proportions and the clinical metadata. **d**, **e** A Kaplan–Meier plot depicting the OS (**d**) and RFS (**e**) of the patients with TNBC in METABRIC after their stratification into the clusters shown in **c** The cluster-5 patients were enriched in *SPP1*^*hi*^ Mφs and showed worse prognosis than the other patients. By contrast, the cluster-3 patients, who were enriched in *CCL3*^*hi*^ Mφs, had a better prognosis, although this difference did not achieve statistical significance.

To confirm the presence of our myeloid subtypes in other TNBC cohorts, we conducted bulk deconvolution on the bulk RNA-seq data of the TCGA and METABRIC cohorts, using BisqueRNA^56^. The METABRIC cohort consists of 1,992 primary breast-cancer samples^83^. Indeed, all neutrophil, monocyte and macrophage subtypes that we identified were detected in both the TCGA and METABRIC datasets. The subtype proportions in the two cohorts and our atlas were generally similar (Fig. 6a, b). Significantly, the *SPP1*^*hi*^ Mφ-enriched samples were associated with worse OS in the TCGA dataset (Supplementary Fig. 8a, b). The patients in the METABRIC dataset were then subjected to hierarchical clustering based on correlations between the monocyte/macrophage subtype proportions (Fig. 6c). This showed stratification of the patients into seven clusters, each represented by enriched monocyte/macrophage subtypes, respectively. This clustering was not affected by clinical status. Strikingly, cluster 5 was enriched in *SPP1*^*hi*^ Mφs and associated with poor OS and relapse-free survival (RFS) (Fig. 6d, e). Moreover, the patients with enriched *SPP1*^*hi*^ Mφs who also harbored *VEGFA*^*hi*^ neutrophils demonstrated worse survival than those with either subtype alone or neither (Supplementary Fig. 8c). By contrast, patients with enriched *CCL3*^*hi*^ Mφs (cluster 3) tended to have a better OS and RFS (Fig. 6d, e). Other patient clusters did not show any significant tendencies (Supplementary Fig. 8d, e). To validate the poor prognosis associated with patients having high levels of angiogenic niche components, we obtained the DEGs of patients with high *SPP1*^*hi*^ Mφs and *VEGFA*^*hi*^ neutrophils from the METABRIC cohort. By scoring these DEGs in TCGA samples, we observed worse prognosis in *SPP1*^*hi*^ Mφ and *VEGFA*^*hi*^ neutrophil enriched patients (Supplementary Fig. 9a). To further strengthen our results, we used BayesPrism, which is a bulk deconvolution tool that has been tested in various tumor samples^59^. BayesPrism results were similar to BisqueRNA results, clearly demonstrating the worse prognosis of *SPP1*^*hi*^ Mφ-enriched patients (Supplementary Fig. 9b, c). A similar stratification was observed when bulk deconvolution was performed using neutrophil subtypes, although the resolution was lower, resulting in clusters with mixed phenotypes (Supplementary Fig. 9d). Nonetheless, bulk deconvolution accurately captured the presence of the myeloid subtypes in other human datasets and distinguished patients on the basis of the proportions of these subtypes. Together, these analyses showed that *SPP1*^*hi*^ Mφs and *VEGFA*^*hi*^ neutrophils in the angiogenic niche have protumoral clinical effects.

### *SPP1*^*hi*^ Mφs are conserved in murine TNBC models and are targetable by cyclophosphamide

**Fig. 7:**
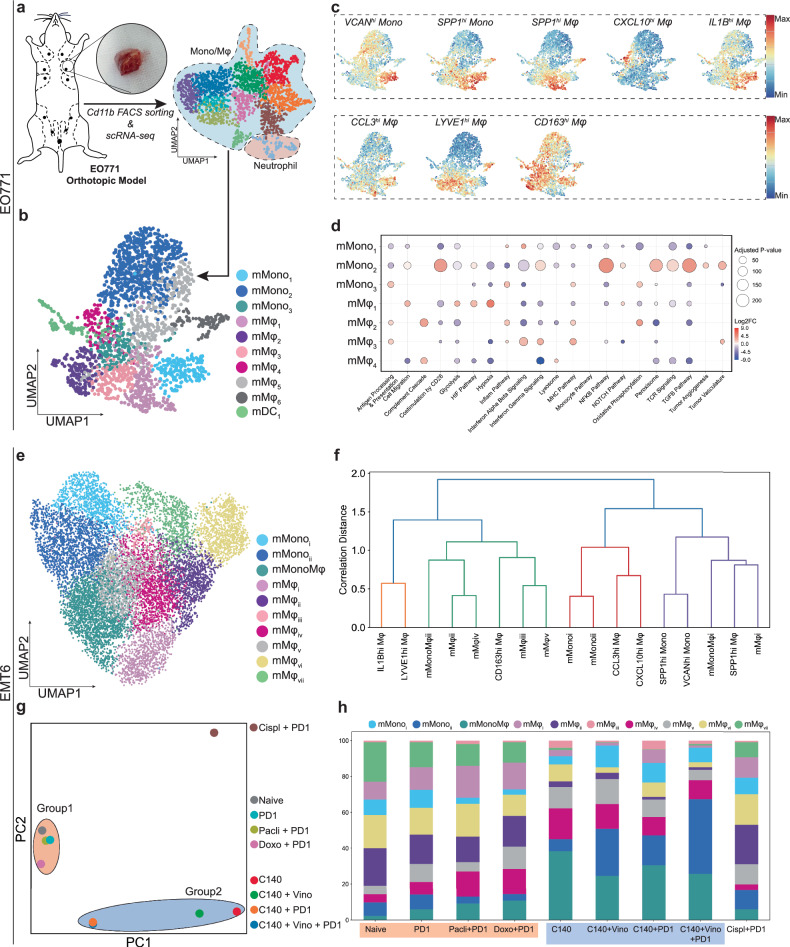
Macrophage subtypes in murine TNBC models overlap with human equivalents. **a** A schematic depiction of the generation of the EO771 orthotopic breast-cancer model and the downstream scRNA-seq analysis. **b** UMAP of the monocytes/macrophages in the EO771 tumors. The monocytes/macrophages clustered into three monocyte subtypes, six macrophage subtypes and one DC subtype. **c** Scoring of the human monocytes/macrophages subtype markers in the EO771 tumors. Only mouse orthologs were used. **d** Pathway activities of the monocytes/macrophages subtypes in the EO771 tumors, as estimated with GSVA. **e** UMAP projection of the monocytes/macrophages sorted from the publicly available EMT6 model (GSE191246). The monocytes/macrophages in the EMT6 tumors subclustered into three monocyte subtypes, seven macrophage subtypes and one intermediate subtype. **f** Hierarchical clustering of the monocyte/macrophage subtypes in human and murine TNBC. The *SPP1*^*hi*^ Mφs are conserved in both humans and mice. **g** A PCA plot of the pseudobulk data derived from scRNA-seq of EMT6 tumors that had been treated with different anticancer regimens. Group 2 is characterized by C140 treatment. **h** The proportion of monocytes/macrophages in the different treatment conditions. *SPP1*^*hi*^ Mφs are decreased after C140 treatment. PD1, αPD-1 ICI; Pacli, paclitaxel; Doxo, doxorubicin; Vino, vinorelbine.

We next asked whether the myeloid subtypes observed in human TNBCs were also observed in murine TNBC models. Thus, a preclinical orthotopic TNBC model was generated by injecting EO771 cells into the second mammary gland of female mice, and scRNA-seq was conducted on the CD11b^+^ myeloid cells in the tumors 33 days later (Fig. 7a). Because few neutrophils were detected, our downstream analysis focused on the monocytes/macrophages. Mapping of the signatures of the monocyte/macrophage subtypes that we identified in human TNBC showed that the murine TNBC contained *SPP1*^*hi*^ Mφs (designated mMφ_1_) (Fig. 7b, c and Supplementary Table 6). The mMφ_1_ were also similar to *SPP1*^*hi*^ Mφs at the functional level because they were enriched in the glycolysis and hypoxia pathways (Fig. 7d).

In addition to the EO771 model, we validated our mapping results in two other murine orthotopic TNBC models, generated using the EMT6 and 4T1 TNBC cell lines, which are commonly used in translational research^84^. For this, the publicly available scRNA-seq data of these models were retrieved^85–87^ (Fig. 7e and Supplementary Fig. 10a). Both models resembled the EO771 model in terms of their correlation with the human data (Supplementary Fig. 10b–d). Strikingly, the mMφ_i_ in the EMT6 model had high *SPP1*^*hi*^ Mφ scores (Supplementary Fig. 10c). Moreover, hierarchical clustering grouped the mMφ_i_ cells together with the human *SPP1*^*hi*^ Mφs and separately from the other monocyte/macrophage subtypes (Fig. 7f). Another notable difference between human and murine TNBC myeloid cells was the presence of nonclassical monocytes (NCMs). While NCMs were barely detectable in human TNBC samples, EO771 models displayed a higher fraction of NCM-like cells (mMono_2_) (Fig. 7b).

The 4T1 and EMT6 scRNA-seq data contained more neutrophils than the in-house EO771 dataset. Mapping of our human neutrophil subtypes showed that most were conserved in the 4T1 and EMT6 murine models (Supplementary Fig. 10e–h).

The public EMT6 dataset that we retrieved consists of nine samples, each with different treatment conditions^85^. We thus asked if any of these treatments influenced the myeloid population in the tumors. Pseudobulk analysis coupled with PCA showed the samples clustered into two treatment groups. The first group consisted of the untreated naive sample and the samples treated with anti-PD-1 alone or in combination with other treatments. Strikingly, the other group included all samples that were treated with cyclophosphamide (C140), either alone or in combination with other therapies (Fig. 7g). This indicates that C140 treatment strongly impacts the intratumoral myeloid environment. Specifically, C140 treatment seemed to increase monocyte populations while reducing several macrophage subtypes, especially the human *SPP1*^*hi*^ Mφ equivalent mMφ_i_. Moreover, paclitaxel treatment was weakly associated with elevated *SPP1*^*hi*^ Mφs (Fig. 7h). These findings suggest that the myeloid environment in murine TNBC resembles that of human TNBC, facilitating translational research on the protumoral *SPP1*^*hi*^ Mφ subtype.

## Discussion

The myeloid cells in the TME have both protumoral and antitumoral functions. The balance depends on the tissue and cancer type^23,39^. Consequently, myeloid cell numbers do not always correlate with outcomes. For example, myeloid cell abundance predicts positive outcomes in colorectal cancer but negative outcomes in breast cancer^25,66,67^. Moreover, we observed that TNBC, which has a poor prognosis compared with other breast cancer subtypes^3^, is associated with higher myeloid infiltration than other subtypes. However, the current understanding of the myeloid population in TNBC remains poor. To address this, we leveraged in-house and publicly available scRNA-seq data to comprehensively characterize the myeloid landscape in TNBCs. Moreover, because the spatial arrangement of immune cells in tumors is clinically significant and in fact has superior prognostic value compared with cell type abundance^63^, we also conducted spatial transcriptomics to determine the spatial distribution and functions in situ. We detected two angiogenic and protumoral myeloid subtypes, namely *SPP1*^*hi*^ Mφs and *VEGFA*^*hi*^ neutrophils. These cells colocalized in a hypoxic and *VEGFA*-rich niche that harbored abundant cancer cells and *APLN*^*hi*^ endothelial tip cells. Our analyses with clinical datasets showed that the abundance of these protumoral subtypes was associated with worse survival in patients with TNBC. Moreover, murine scRNA-seq analyses revealed the presence of conserved protumoral *SPP1*^*hi*^ Mφs in murine TNBC, thus opening avenues for translational research.

The colocalization of *SPP1*^*hi*^ Mφs with cancer cells in a hypoxic region and the association of *SPP1*^*hi*^ Mφs with poor prognosis has also been observed in other cancers, including hepatocellular carcinoma, colorectal carcinoma and head-and-neck squamous carcinoma^25,27,88^. This is consistent with what we observed in TNBC, which has not yet been reported in this particular deadly cancer type.

We discovered that *SPP1*^*hi*^ Mφs did not localize near established vessels, explaining their upregulation of hypoxia- and metabolic stress-related pathways such as glycolysis, adipogenesis and cholesterol homeostasis. Moreover, *SPP1*^*hi*^ Mφs respond to the hypoxic niche by promoting angiogenesis: they display a strong angiogenesis-related transcriptomic signature, express large amounts of ADM and are in close proximity with *APLN*^*hi*^ endothelial-tip cells that express the CALCRL receptor for ADM. This is consistent with Wang et al., whose analysis of glioblastoma revealed a hypoxia TAM that produced ADM and destabilized endothelial adherens junctions, thus creating a hyperpermeable vasculature that associates with worse prognosis^79^. Pan et al. also showed that *APLN*^*hi*^ endothelial-tip cells promote cancer progression and poor prognosis in multiple cancer types^78^. In addition, we found that *VEGFA*^*hi*^ neutrophils colocalized with the *SPP1*^*hi*^ Mφs and *APLN*^*hi*^ tip cells in the hypoxic region. Notably, we observed that, while higher numbers of *SPP1*^*hi*^ Mφs associated with poor survival in TNBC, this was greatly worsened when the patients also harbored high numbers of *VEGFA*^*hi*^ neutrophils. These observations together suggest that ADM-producing *SPP1*^*hi*^ Mφs and VEGF-producing *VEGFA*^*hi*^ neutrophils create a proangiogenic environment that promotes the generation of endothelial-tip cells, which in turn reduce patient survival in TNBC. This suggests that therapeutically targeting one or more of these elements in the hypoxic niche could normalize tumor vessels, thereby improving TNBC outcomes. This approach is supported by Wang et al., who showed that pharmacological blockade of ADM restores vascular integrity in glioblastoma and improves intratumoral concentrations of an antitumor agent^78,79^.

The colocalization of *VEGFA*^*hi*^ neutrophils and *SPP1*^*hi*^ Mφs and the fact they both had angiogenic functions suggested that they may be generated by the same environmental cue-induced transcription factor. Indeed, our regulon analysis showed that ATF4 was activated in both myeloid subtypes and in the hypoxic angiogenic niche (region cluster 1). ATF4 has been reported to be one of the master regulators of cellular stress, especially endoplasmic reticular stress^89^. Specifically, severe and prolonged hypoxia, which is found inside solid tumors, leads to accumulation and aggregation of misfolded proteins, resulting in endoplasmic reticular stress and, thereby, the activation of ATF4. Furthermore, hypoxic factors have been reported to directly activate ATF4. For instance, hypoxia induces the phosphorylation of eukaryotic initiation factor 2 alpha subunit (eIF2a), which leads to upregulation of ATF4^90–92^. ATF4, upregulated via hypoxia, can promote angiogenesis via VEGFA signaling^93^ or modulation of extracellular matrix organization^94^. Furthermore, ATF4 has been reported to prevent proteasomal degradation of HIF1a, which is another major player in cellular response to hypoxia. Notably, Wang et al. showed that their hypoxia TAM in glioblastoma was driven by *HIF1A* along with p50 (*NFKB1*) and c-Jun^79^. Similarly, the *SPP1*^*hi*^ Mφs identified by Fan et al. in hepatocellular carcinoma were regulated by *HIF1A*^88^, as was the TAM2 subtype that Dunsmore et al. identified in pancreatic adenocarcinoma^95^. Thus, hypoxia-driven ATF may induce the angiogenic functions in both *VEGFA*^*hi*^ neutrophils and *SPP1*^*hi*^ Mφs in TNBC, possibly by activating HIF1α expression. However, the precise mechanism by which hypoxia activates ATF4 and how ATF4 is involved in the reprogramming of myeloid cells in TNBC remain to be determined. In addition, although our results demonstrate activation of ATF4, other transcription factors may also contribute to the development of both *VEGFA*^*hi*^ neutrophils and *SPP1*^*hi*^ Mφs. For instance, in pancreatic cancer, BHLHE40 has been identified as a key regulator of angiogenic neutrophil development^28^. These findings suggest that the development of angiogenic myeloid subtypes may be governed by the coordinated action of multiple transcription factors, rather than a single factor.

Murine tumor models are crucial for translational research, and several studies have sought to compare the human and murine cell types in various cancers^18,22^. Mapping the signatures and functions of human myeloid subtypes in three well-known murine TNBC models (EO771, 4T1 and EMT6), revealed the conservation of *SPP1*^*hi*^ Mφs and most neutrophil subtypes in the murine TNBCs. This result also aligns with a previous study by Zilionis et al., which reported the conservation of neutrophils and the differences of macrophage subtypes across species^18^. Another notable difference across species was the detection of NCM-like cells in murine EO771 tumors. While human TNBC samples contained very few NCM-like cells, EO771 tumors exhibited a relatively high accumulation of these cells (mMono_2_). Moreover, our analysis of the EMT6 data showed that TNBC treatment with C140 was associated with reduced *SPP1*^*hi*^ Mφ frequencies. C140 treatment has been reported to reduce the number of peripheral blood leukocytes, including myeloid cells^96^, which might be one reason for the smaller number of *SPP1*^*hi*^ Mφs in murine models. However, other reports exist in which C140 treatment induced the development of immune-suppressive myeloid subtypes^97,98^, indicating that a detailed analysis of the impact of therapeutic drugs on the myeloid environment in human and murine TNBC is warranted. As EMT6 has also been reported as an ER-positive breast cancer cell line^99^, we validated our findings in other murine TNBC models to provide a thorough explanation in the context of TNBC.

It is notable that we were able to capture neutrophils in our scRNA-seq analysis. These cells are often underrepresented in scRNA-seq data because of their fragility, short lifespan and very low mRNA levels. This means that their subtypes and roles in cancer have been less robustly characterized compared with other immune cell types. However, recent studies have made new strides in the field^43,52,100–103^. They include the scRNA-seq study by Wu et al.: their analysis of 17 cancer types showed, like our study, that the cancer-associated neutrophils are extraordinarily diverse and complex. However, that study combined several cancer types and did not distinguish between TNBCs and non-TNBCs^52^. Salcher et al. also showed that the neutrophils in non-small cell lung cancer are a major source of *VEGFA* expression in the TME and are associated with anti-PDL1 treatment failure^43^. Moreover, Ng et al. found that some TME neutrophils in an animal model of pancreatic cancer promoted angiogenesis and that depleting them or inhibiting their angiogenic function reduced tumor growth^41^. By analyzing neutrophil subtypes along with their developmental trajectories and functions in TNBC, our study provides valuable insights and contributes significantly to this emerging field.

Our study has some limitations. Because the neutrophils in our atlas were derived mainly from in-house samples, individual patients varied in terms of neutrophil subtype abundance. Such interpatient myeloid cell heterogeneity has been reported in previously research^23,39^. Thus, further investigations on why there is such heterogeneity are needed. Moreover, our study is limited by the technical limitations of the scRNA-seq technique. Some analyses of this study are based on bulk deconvolution, a technique with recognized limitations^104^. To enhance the robustness of our findings, we applied two independent algorithms—BisqueRNA and BayesPrism—for cross-validation. Nevertheless, additional validation using orthogonal methods such as flow cytometry may be warranted.

In summary, our study provides insights into the previously unexplored myeloid milieu in TNBC. In particular, we demonstrate that angiogenic myeloid cell subtypes reside in a hypoxic niche in proximity to *APLN*^*hi*^ tip cells and correlate with poor outcomes in patients with TNBC. By demonstrating that these myeloid cell subtypes are also present in mouse models of TNBC, this study lays a foundation for further translational research.

## Supplementary information

Supplementary Information

Supplementary Table 1

Supplementary Table 2

Supplementary Table 3

Supplementary Table 4

Supplementary Table 5

Supplementary Table 6

Supplementary Table 7

## Data Availability

All data needed to evaluate the conclusions of this study are present in the Article and/or the supplementary tables. Raw and processed data from the scRNA-seq experiments conducted in this study have been deposited in the NCBI Gene Expression Omnibus (GEO) under accession number GSE284115, or are accessible through their original publications^26,34,85^. Spatial transcriptomics data are available from their original publication^60^.
