# Supplementary material for: Single-cell transcriptomics of the myeloid milieu reveals an angiogenic niche in triple-negative breast cancer
Source: Exp Mol Med. 2025 Nov 7;57(11):2487–504. doi: 10.1038/s12276-025-01571-5 (PMC12686459; doi:10.1038/s12276-025-01571-5)
Supplement: Supplementary file 1 — Supplementary Information [file 12276_2025_1571_MOESM1_ESM.pdf]

Supplementary Materials for

Single-cell transcriptomics of myeloid milieu reveals an angiogenic niche in triple-negative breast cancer

Yechan Choi, Minkyu Shim *et al.*

*Corresponding author. Keehoon Jung, keehoon.jung@snu.ac.kr;
Han-Byoel Lee, hblee.md@snu.ac.kr;
Charles Lee, charles.lee@jax.org

This PDF file includes:

Supplementary Figs. 1 to 10
Legends for Supplementary Tables 1 to 7

Other Supplementary Material for this manuscript includes the following:

Supplementary Tables 2 to 7

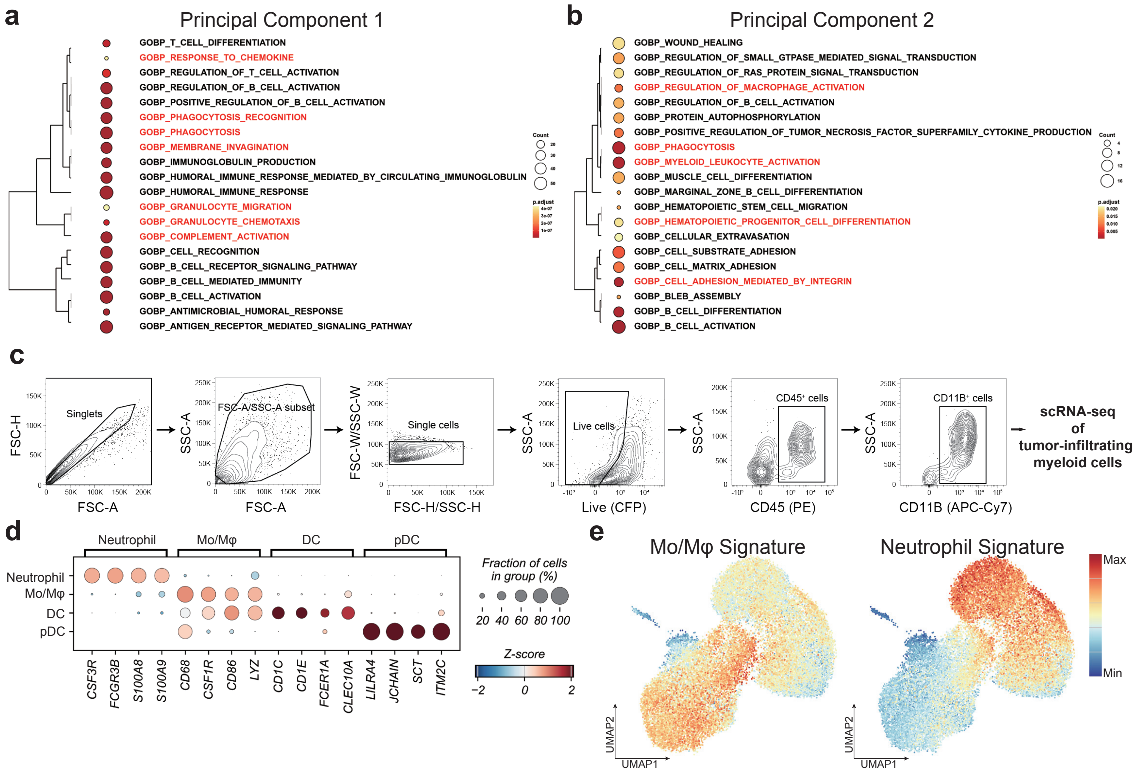

Supplementary Fig. 1. Importance of myeloid cells in TNBCs and construction of a TNBC myeloid-cell scRNA-seq atlas.

a, b, Bulk RNA-seq data of the TNBCs and non-TNBCs in the TCGA-BRCA cohort were subjected to PCA and GO analysis of the top 200 genes from **(a)** PC1 and **(b)** PC2. PC1 and PC2 are enriched in myeloid function-related genes (red). **c-e,** Construction of our TNBC myeloid-cell scRNA-seq atlas. **c,** Myeloid cells from primary TNBCs were isolated with FACS sorting for Singlet-Live-CD45+-CD11b+ cells. **d,** Expression of the following canonical myeloid markers by the high-quality myeloid cells: *CSF3R, FCCGR3B, S100A8, S100A9* (neutrophils); *CD68, CSF1R, CD86, LYZ* (monocytes/Mφ); *CD1C, CD1E, FCER1A, CLEC10A* (DCs); *LILRA4, JCHAIN, SCT, ITM2C* (pDCs). **e,** Feature plot depicting the score of the cell-type signatures sourced from PanglaoDB.

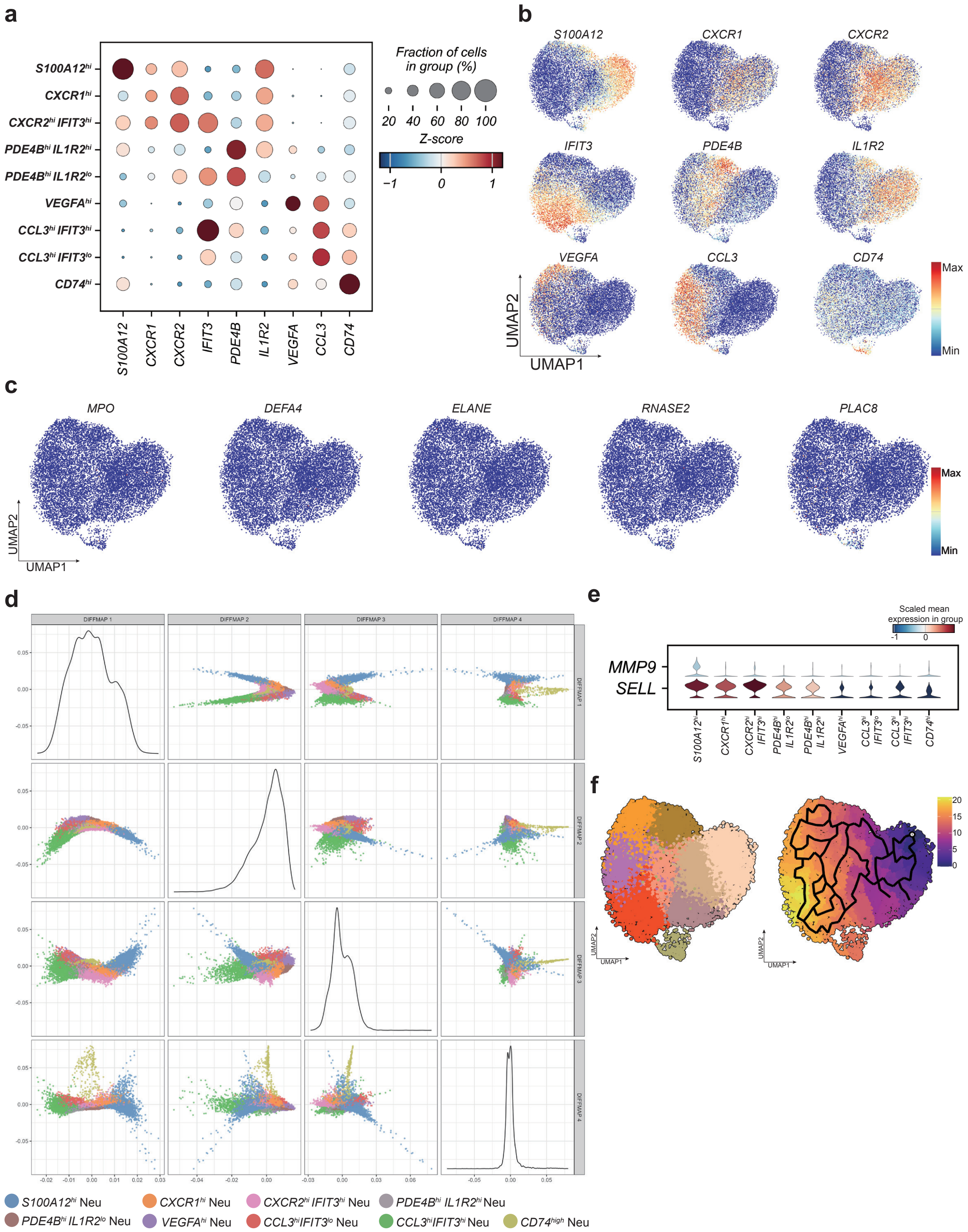

Supplementary Fig. 2. Neutrophils display diverse developmental trajectories and functions.

a, Dot plot visualizing the marker expression that was used to define the neutrophil subtypes. The color and size represent the mean expression and the fraction of cells in each group, respectively. **b,** Feature plot depicting the marker expression of each neutrophil subtype. **c,** Feature plot illustrating the marker expression of immature neutrophils. These

markers were not expressed in any of the neutrophil subtypes. **d,** Two-dimensional visualization of neutrophils using different combinations of diffusion components. **e,** Stacked violin plot depicting the expression of *MMP9* and *SELL* in the neutrophil subtypes. *MMP9* and *SELL* are mainly enriched in *S100A12*^{hi} neutrophils. **f,** The trajectory map of the neutrophil subtypes that was constructed with Monocle 3. *S100A12*^{hi} neutrophils was observed to differentiate continuously, ending in the *CCL3*^{hi} *IFIT3*^{lo} neutrophils.

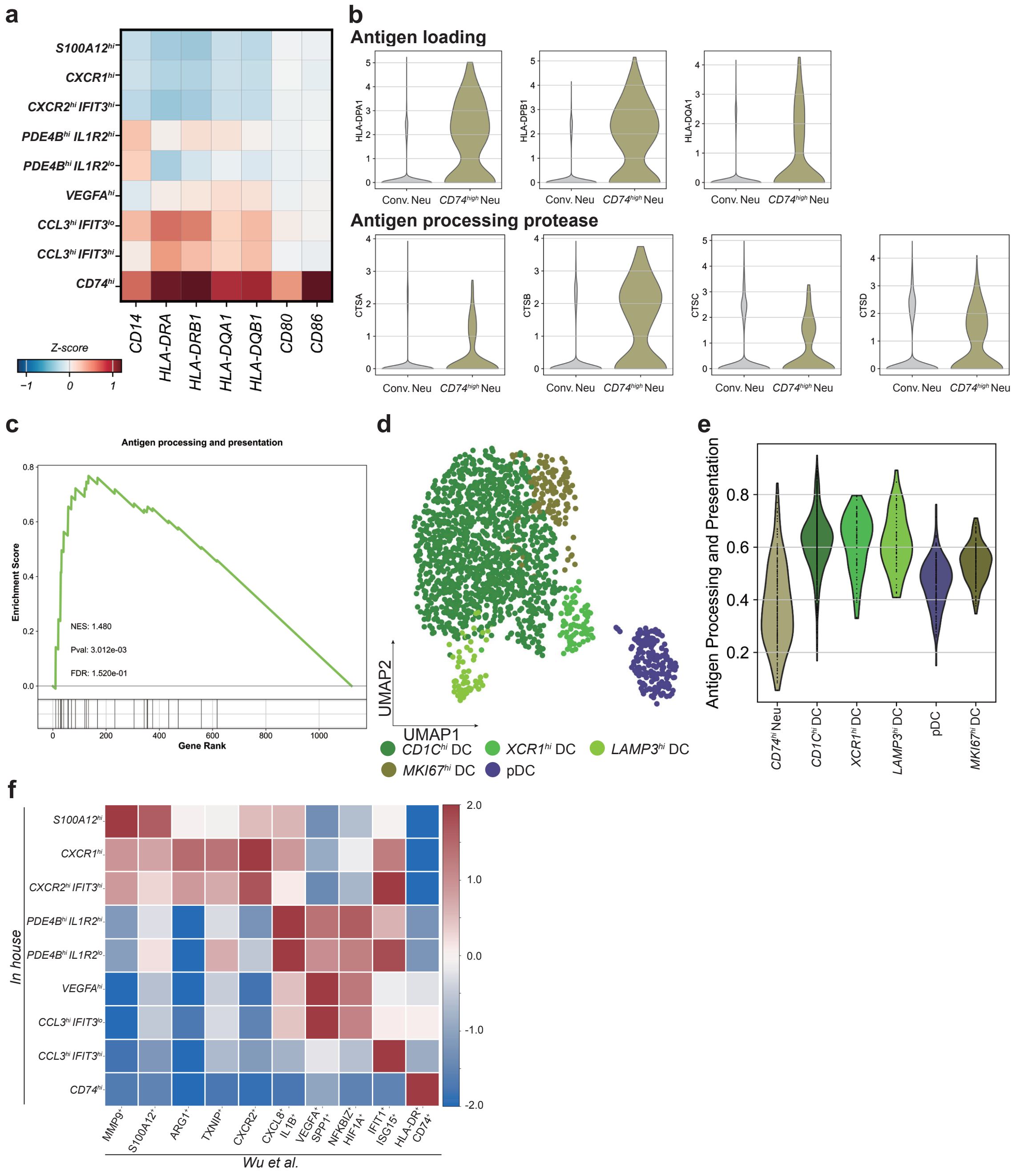

Supplementary Fig. 3. *CD74^{hi}* neutrophils bear antigen-presenting capability, although it is less potent compared to in dendritic cells.

a, Matrix plot depicting the expression of antigen-presenting neutrophil markers by the nine neutrophil subtypes. The markers were previously used to characterize the antigen-presenting neutrophil subtype that was detected in early lung cancer by Singhal et al.[72]. **b,** Violin plot illustrating *CD74^{hi}* neutrophil expression of genes that encode antigen-loading proteins or antigen-processing proteases. The *CD74^{hi}* neutrophils expressed these proteins

and proteases at higher levels than conventional neutrophils. **c,** GSEA plot of the antigen-processing and -presentation pathway in *CD74^hi^* neutrophils. **d,** UMAP of the DC landscape in TNBC. **e,** Violin plot comparing the scores of the KEGG gene set: Antigen Processing and Presentation (hsa04612) of the $CD74^{hi}$ neutrophils and DC subtypes. The $CD74^{hi}$ neutrophils have lower scores than the DC subtypes. **f,** Matrix plot comparing the signatures of the neutrophil subtypes in the present study to the previously published signatures of the neutrophils that were detected by pan scRNA-seq of 17 cancers[51]. DC: dendritic cell.

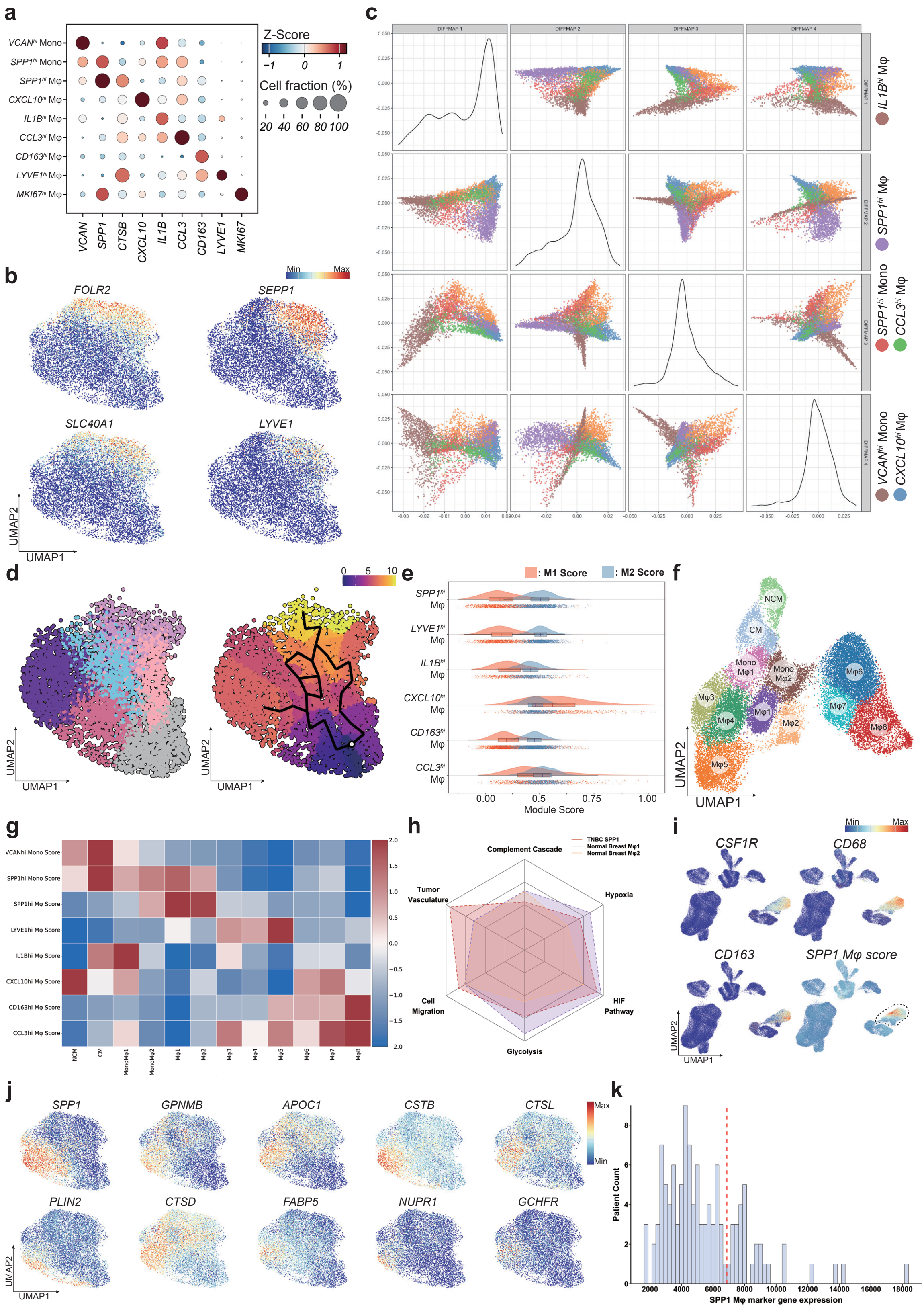

Supplementary Fig. 4. Monocytes and macrophages display distinct and functionally disparate lineages.

a, Dot plot depicting the marker expression of the monocyte and macrophage subtypes. The size and color indicate the z-score and cell fraction, respectively. **b,** Feature plot illustrating TRM marker expression. The TRM markers are localized in the *LYVE1*^{hi} Mφs and *CD163*^{hi} Mφs. **c,** Two-dimensional visualization of the differentiation of monocytes and monocyte-derived macrophages, as determined with different combinations of diffusion components. **d,** Differentiation trajectories were mapped on the UMAP of monocytes and monocyte-derived macrophages by using Monocle 3. Two lineages that ended in *SPP1*^{hi} Mφs and *CCL3*^{hi} Mφs were observed. **e,** Rain cloud plot portraying the M1 and M2 scores of the macrophage subtypes. The *SPP1*^{hi} Mφs have a low M1 score. **f,** UMAP of the monocyte/macrophage subtypes in normal breast tissues. The scRNA-seq data were retrieved from a previous publication[33]. **g,** Matrix plot depicting the DEG scores of the TNBC subtypes relative to the normal breast-tissue subtypes. **h,** Radar plot depicting the pathway activities of *SPP1*^{hi} Mφs, Mφ1, and Mφ2, as calculated by using AUCell. The *SPP1*^{hi} Mφs showed heightened tumor-vasculature pathway activity. **i-k,** A combined dataset consisting of the TNBC dataset from Wu et al.[26] and the myeloid population in the present study was generated. **i,** Feature plot depicting the *SPP1*^{hi} Mφ-marker scores as well as macrophage marker genes in the combined dataset. The *SPP1*^{hi} Mφ signature localized to the monocytes and macrophages. **j,** Feature plot depicting the top 10 *SPP1*^{hi} Mφ DEGs in the monocytes and macrophages. **k,** Histogram illustrating the distribution of *SPP1*^{hi} Mφ-marker expression in the TNBC patients in the TCGA cohort. The red dashed line indicates the cutoff point used for stratification.

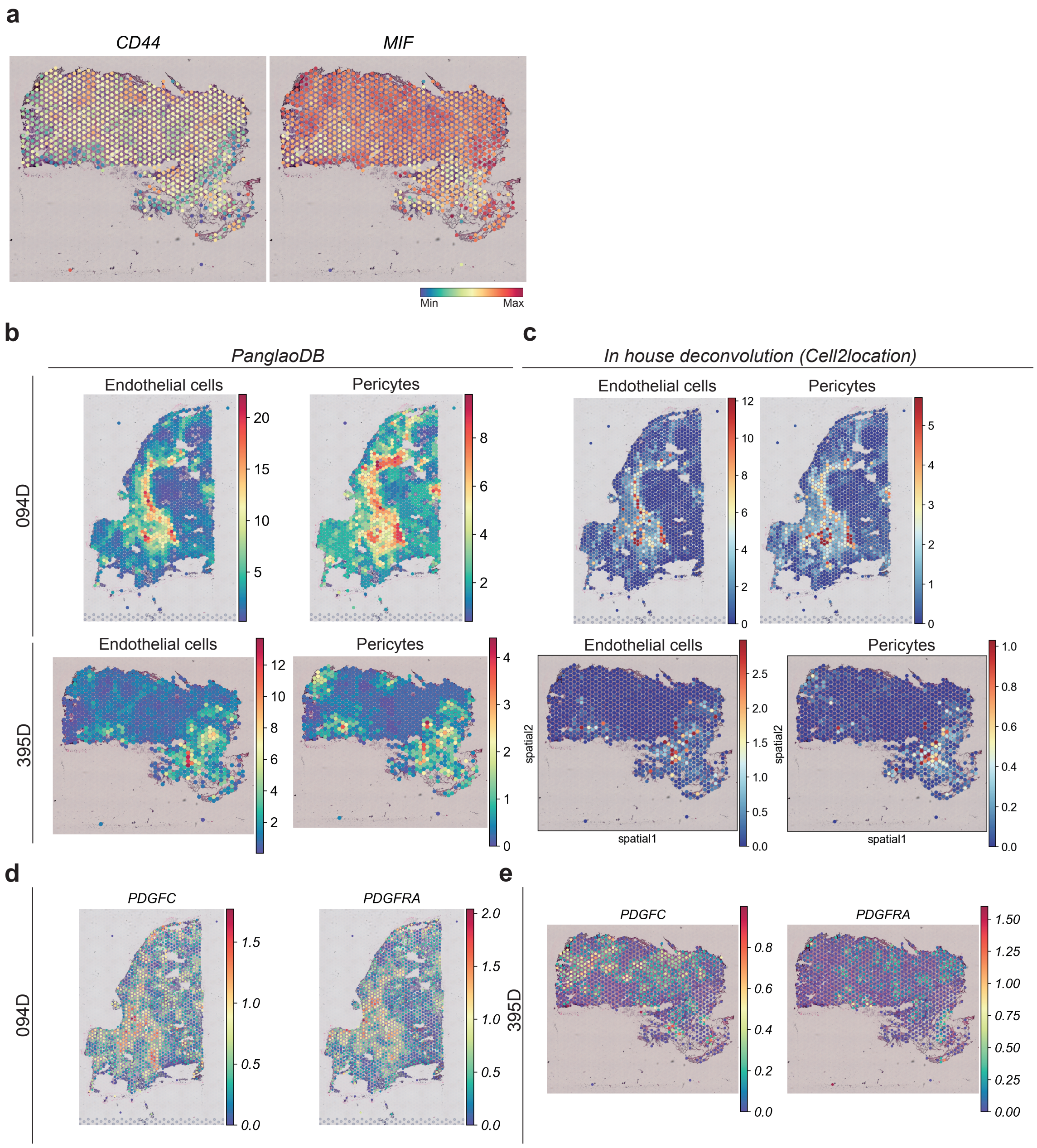

Supplementary Fig. 5. Spatial deconvolution shows that the classical lineage and TRM macrophages reside at blood vessels.

a, Spatial expression of *CD44* and *MIF* in 395D. *CD44* and *MIF* are highly expressed in the hypoxic regions that are enriched with *SPP1*^{hi} Mφs and epithelial cells. **b, c,** The spatial distribution of endothelial cells and pericytes in 094D and 395D, estimated by using markers from PanglaoDB **(b)** and deconvolution with our scRNA-seq atlas **(c)**. Endothelial cells and

pericytes are localized in regions that are enriched with classical lineage and TRMs. **d, e,** Spatial expression of *PDGFC* and *PDGFRA* in **(d)** 094D and **(e)** 395D.

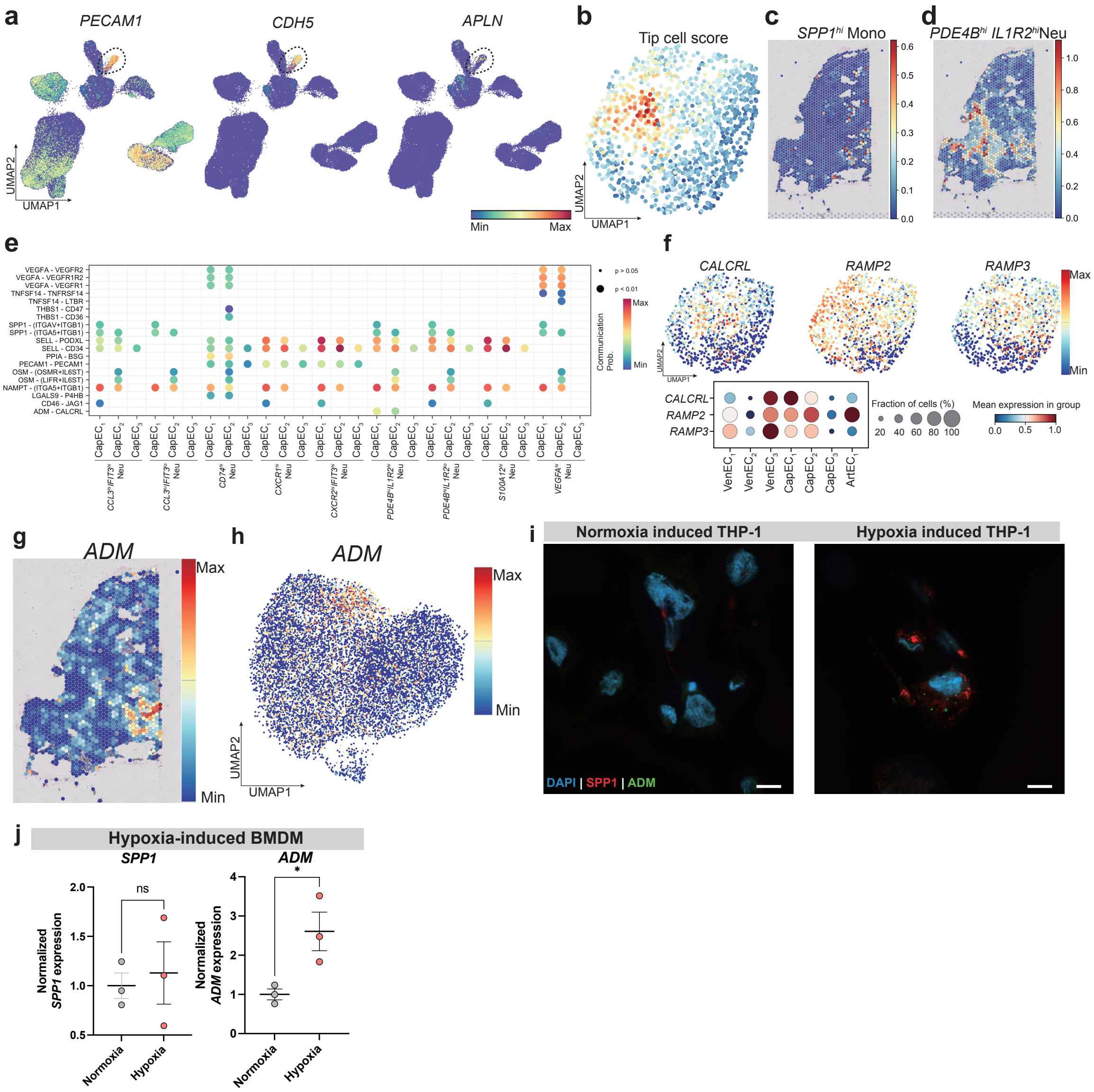

Supplementary Fig. 6. Delineation of the EC subsets in TNBCs and regulon-activity analysis.

a, Feature plot depicting *APLN*, PECAM1, and CDH5 expression in our atlas. Areas encircled with dashed lines indicate ECs. *APLN* is mainly expressed in ECs. **b,** Feature plot illustrating the tip-cell score in our atlas. Scoring was conducted using the markers described by Pan et al.[76]. **c,d,** Spatial location of *SPP1*^{hi} monocytes **(c)** and *PDE4B*^{hi} *IL1R2*^{hi} neutrophils **(d)** in the representative Visium slide 095D. **e,** Bubble plot showing significant ligand-receptor pairs between neutrophil subtypes and capillary-EC subtypes, including CapEC$_1$, which contains the tip cells. *VEGFA*^{hi} neutrophils actively interact with CapEC$_1$. **f,** ADM-receptor expression in the EC subtypes. Of the three capillary EC subtypes, CapEC1

exhibited the greatest receptor expression. **g,** ADM expression on the representative slide 094D. **h,** *ADM* expression in neutrophils. **i,** SPP1 and ADM expression in normoxia or hypoxia induced THP-1 cells. Scalebar: 5 μm. **j,** Expression of SPP1 and ADM in hypoxia treated BMDMs. **g,** Spatial location of Region cluster 1 in slide 095D.

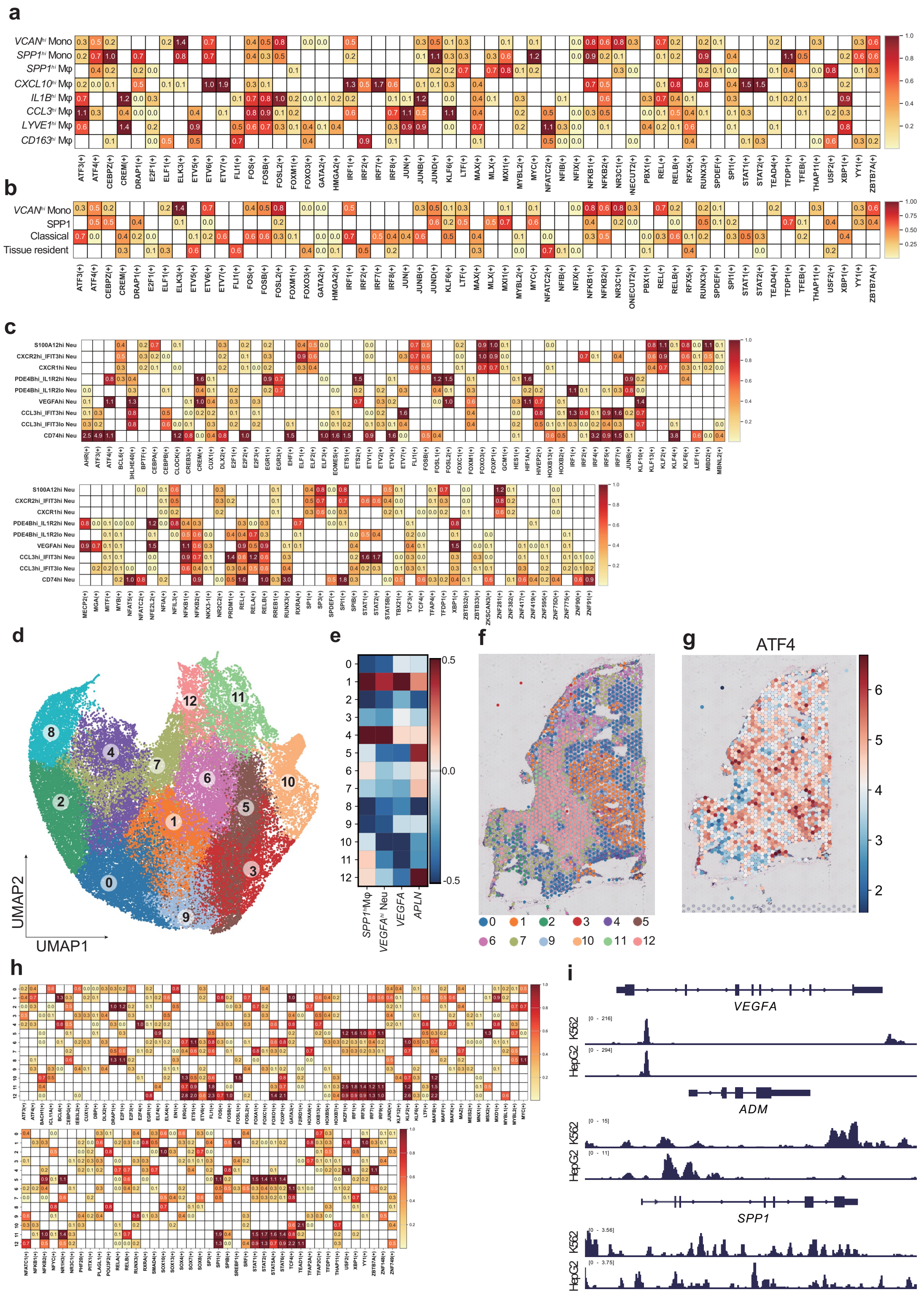

Supplementary Fig. 7. ATF4 is a major regulator of the angiogenic niche component cells. a-b, pySCENIC-inferred regulon activity in **(i)** the monocyte/macrophage subtypes and **(j)** the *VCAN*^{hi} monocytes, the SPP1-lineage cells, the classical-lineage cells, and the TRMs.

c, Regulon activity inferred by pySCENIC in neutrophils subtypes. **d,** The Visium spots of all slides were clustered according to their cell-type proportion, as inferred with cell2location. **e,** Analysis of the *SPP1*^{hi} Mφ and *VEGFA*^{hi} neutrophil abundance and *VEGFA* and *APLN* expression in the Region clusters in **(d)**. Region cluster 1 is enriched in *SPP1*^{hi} Mφs and *VEGFA*^{hi} neutrophils and expresses high levels of *VEGFA* and *APLN*. **f,** Distribution of Visium spot clusters from (d) on a representative slide (094D). **g,** Inference of ATF4 activity in a representative slide (094D) using decoupleR. ATF4 activity is upregulated in the angiogenic niche. **h,** Regulon activity inferred by pySCENIC in the Region clusters. **i,** Genome browser view depicting the ATF4 binding regions in VEGFA, ADM, SPP1 genome regions.

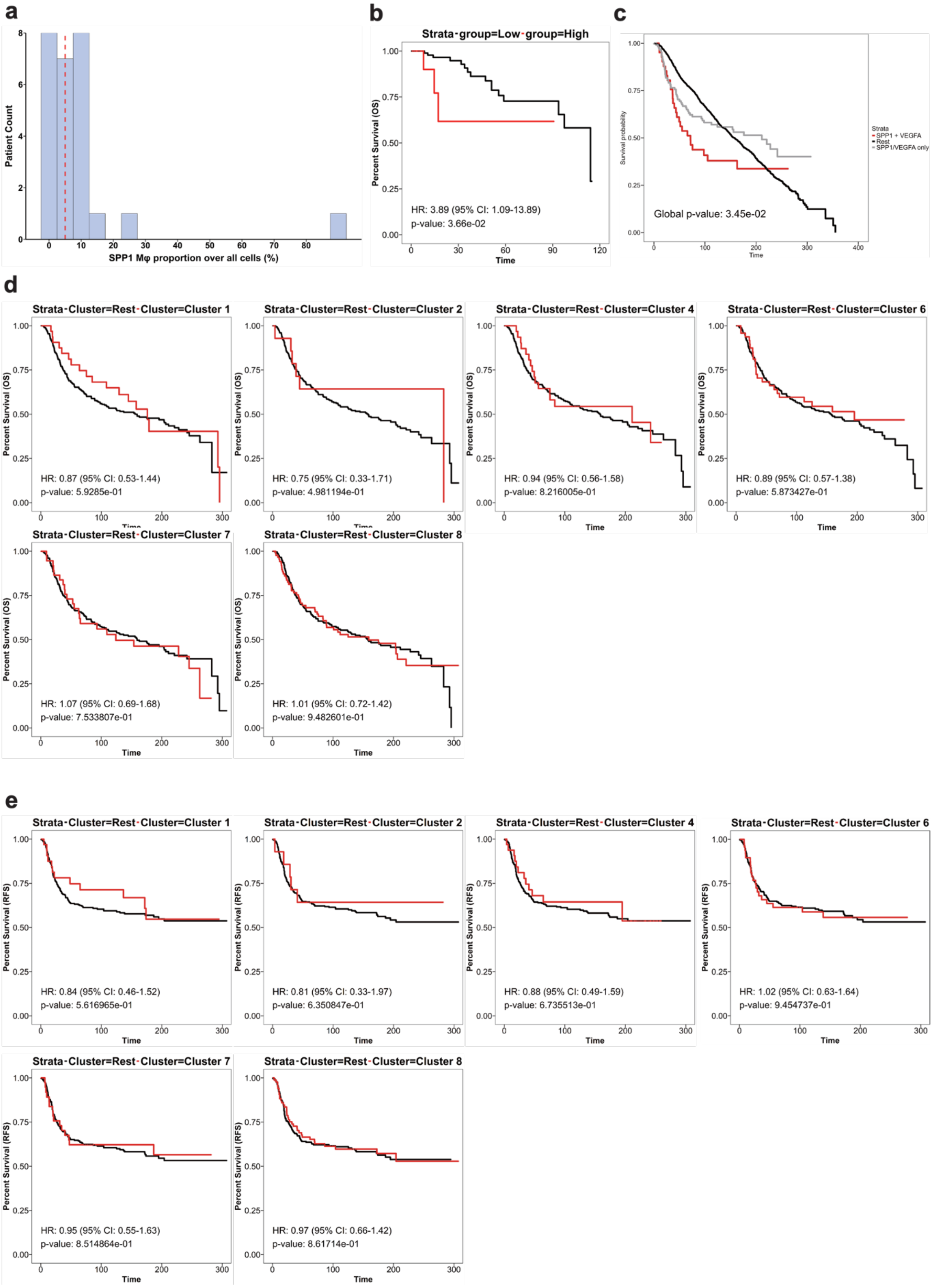

Supplementary Fig. 8. Survival analysis of TNBC patients after their myeloid subtype composition. a, Histogram depicting the distribution of *SPP1*^{hi} Mφ proportions in the TNBC

patients in TCGA. The red dashed line indicates the cutoff point for stratification. **b,** Kaplan-Meier plot illustrating the overall survival of patients stratified by *SPP1*^{hi} Mφ proportion. Patients with high *SPP1*^{hi} Mφs proportion had negative outcomes. The *p*-value was calculated using Cox regression. **c,** Kaplan-Meier plot demonstrating the survival difference of the TNBC patients in METABRIC after stratification according to *SPP1*^{hi} Mφ and *VEGFA*^{hi} neutrophil proportion. Patients containing both *SPP1*^{hi} Mφs and *VEGFA*^{hi} neutrophils had significantly worse prognosis. **d,e,** Kaplan-Meier plot depicting the **(d)** overall survival and **(e)** relapse-free survival of the remaining TNBC patient clusters of **Fig. 6c**.

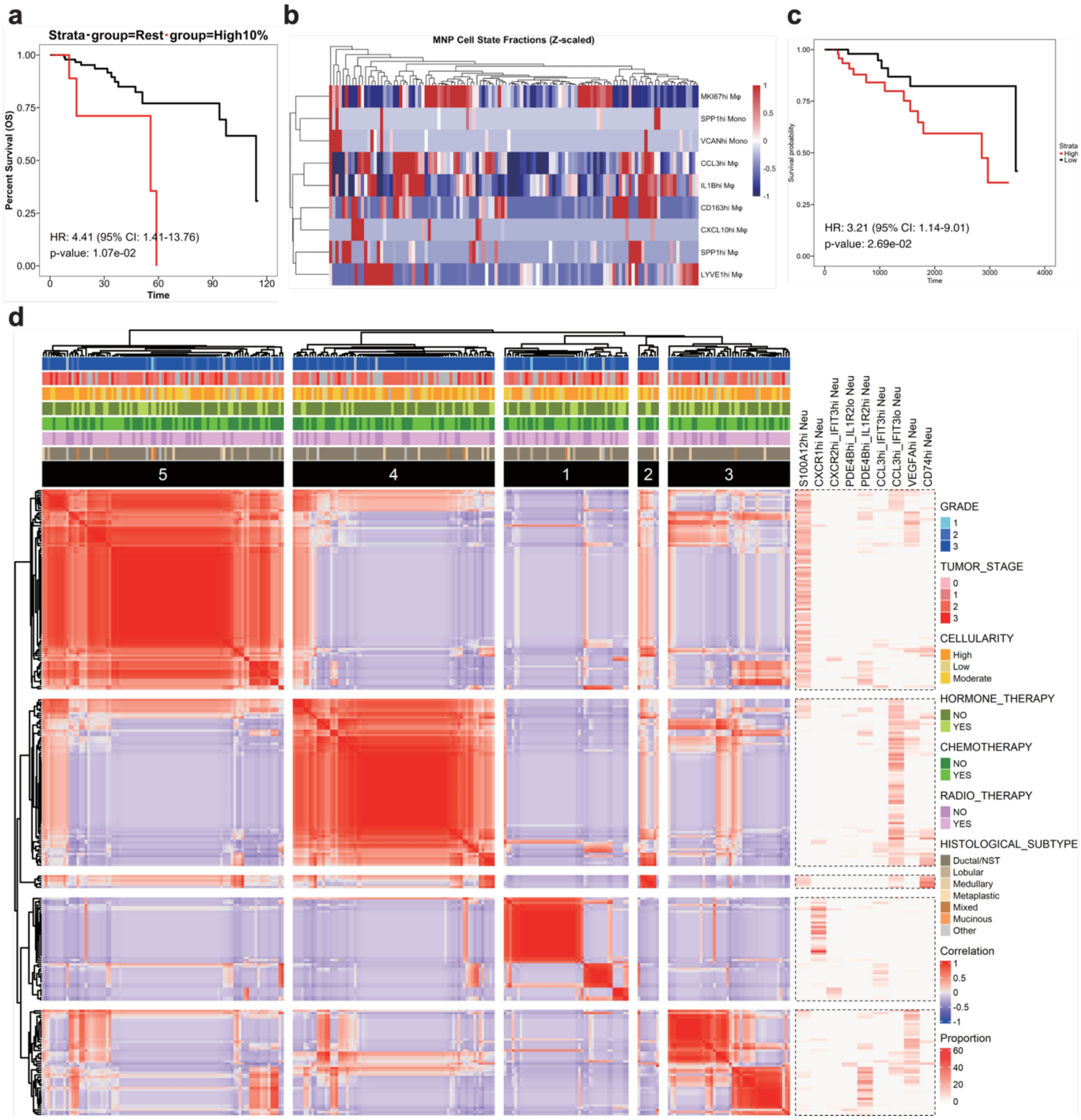

Supplementary Fig. 9. Bulk deconvolution stratifies patients on the basis on neutrophil cell-subtype proportion and shows that angiogenic niche-enriched subtypes associate with worse clinical outcomes. a, Kaplan-Meier plot showing the overall survival of TNBC patients in the TCGA-BRCA cohort stratified by enrichment of *SPP1*^{hi} Mφ and *VEGFA*^{hi} neutrophil signatures. The gene signature defining *SPP1*^{hi} Mφ and *VEGFA*^{hi} neutrophil enrichment was derived from the METABRIC cohort **(Fig. 6c)** and applied to the TCGA cohort. Patients were grouped into “high” (top 10%) and “low” (bottom 90%) clusters based on their signature scores. **b,** MNP fractions in each TNBC patient of the

TCGA cohort, deconvoluted using BayesPrism. **c,** Kaplan-Meier plot depicting the overall survival of TNBC patients, stratified by their *SPP1*^{hi} Mφ abundancies, inferred by BayesPrism. **d,** Hierarchical clustering of the TNBC patients in the METABRIC cohort on the basis of correlations between the neutrophil-subtype proportion.

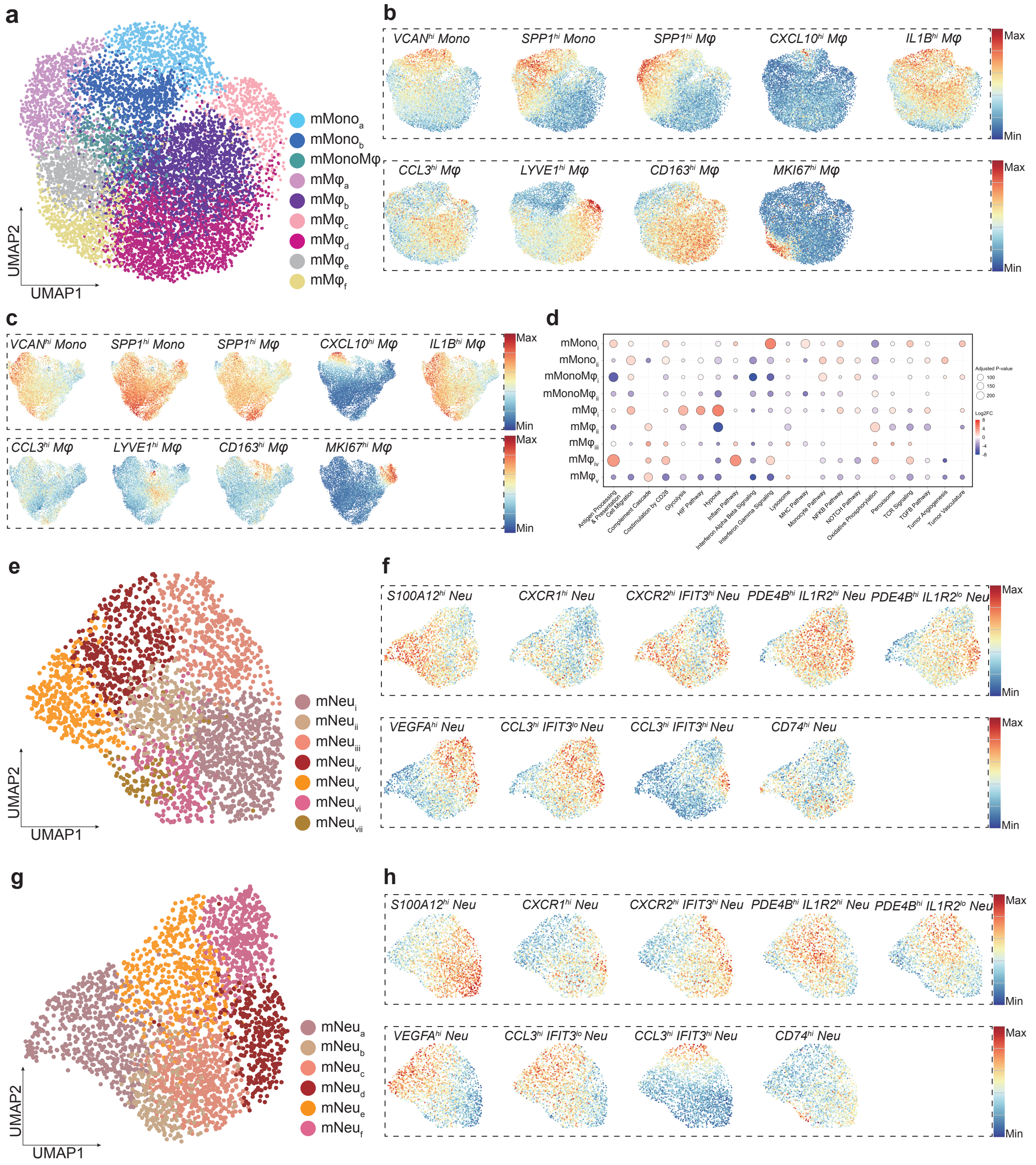

Supplementary Fig. 10. Cross-species comparison of human-myeloid subtypes in murine TNBC models.

a, UMAP of the monocytes and macrophages in the murine 4T1 model. **b,** Feature plot of the scores based on human monocyte/macrophage-subtype DEGs that were mapped to 4T1. **c,** Feature plot depicting the score based on human monocyte/macrophage-subtype DEGs that were mapped to EMT6. *SPP1*^{hi} Mφs are conserved across species. **d,** Dot plot illustrating the enriched pathways in the monocyte/macrophage subtypes that were derived

from EMT6. mMφi shows similar pathways as *SPP1*^{hi} Mφs, including glycolysis and hypoxia. **e,** UMAP of neutrophils in the murine 4T1 model. **f,** Feature plot depicting the scores based on human neutrophil subtype DEGs that were mapped to 4T1. **g,** UMAP of the neutrophils in the murine EMT6 model. **h,** Feature plot depicting the score that was calculated by using DEGs from human neutrophil subtypes and then mapped to EMT6 neutrophils.

Supplementary Table 1. Patient cohort details.

File: Supplementary Table 1.xlsx

Clinical and pathology details for breast cancer patients analyzed by scRNA-seq in this study. ER: estrogen receptor; PR: progesterone receptor; human epidermal receptor 2; IHC: immunohistochemistry; IDC: infiltrating ductal carcinoma; TNBC: triple negative breast cancer; BCS: breast-conserving surgery; SLNB: sentinel lymph node biopsy.

Supplementary Table 2. Genes used for analyzing geneset enrichment in myeloid subsets.

File: Supplementary Table 2.xlsx

Supplementary Table 3. Cell type markers for the identification myeloid cell types.

File: Supplementary Table 3.xlsx

Supplementary Table 4. Differentially expressed genes across neutrophil subclusters.

File: Supplementary Table 4.csv

Supplementary Table 5. Differentially expressed genes across monocyte, macrophage subclusters.

File: Supplementary Table 5.csv

Supplementary Table 6. Differentially expressed genes across myeloid cell subclusters in murine TNBC models.

File: Supplementary Table 6.xlsx

Supplementary Table 7. List of primers used in this study.

File: Supplementary Table 7.xlsx
